# Selective cargo sorting in stem cell‐derived small extracellular vesicles: impact on therapeutic efficacy for intervertebral disc degeneration

**DOI:** 10.1002/ctm2.1494

**Published:** 2023-11-30

**Authors:** Zhiwei Liao, Bide Tong, Xiaoguang Zhang, Weifeng Zhang, Wencan Ke, Huaizhen Liang, Ming Lei, Wenbin Hua, Shuai Li, Yu Song, Xinghuo Wu, Cao Yang

**Affiliations:** ^1^ Department of Orthopaedics Union Hospital Tongji Medical College Huazhong University of Science and Technology Wuhan China

**Keywords:** extracellular vesicles, ferroptosis, intervertebral disc regeneration, miRNA sorting, oxidative stress

## Abstract

**Background:**

Growing evidence has suggested the role of stem cell‐derived small extracellular vesicles (sEVs) in intervertebral disc degeneration (IVDD). The cargo sorting of sEVs, particularly miRNAs, may be influenced when the donor cell is subjected to oxidative stress. Here, we discovered that miRNAs containing specific motifs are selectively sorted into intraluminal vesicles within mesenchymal stem cells (MSCs) in response to oxidative stress.

**Methods:**

Analysis of miRNA cargoes in sEVs derived from normal MSCs (C‐sEVs) or stressed MSCs (T‐sEVs) was conducted using miRNA sequencing. Differential expressed miRNAs in sEVs and the identification of motifs were evaluated through bioinformatics analysis. Protein binding was assessed using immunofluorescent staining and immunoprecipitation analysis. Additionally, RNA pull down and RNA immunoprecipitation (RIP) immunoprecipitation were employed to determine the binding between miRNAs and proteins. The effects of C‐sEVs and T‐sEVs on IVDD were compared by detecting the expression levels of phenotypic genes in vitro or histological evaluation in vivo.

**Results:**

The sorting process of miRNAs is mediated by the nucleocytoplasmic transport of heterogeneous nuclear ribonucleoproteins, which in turn facilitates the phosphorylation of SNAP25 and promotes the transport and secretion of sEVs. Additionally, CHMP1B plays a role in membrane repair and protects against cell ferroptosis upon oxidative stress, concurrently affecting the release of sEVs. Notably, stem cell‐derived sEVs associated with ferroptosis impair the therapeutic efficacy for IVDD. However, the application of engineered sEVs containing a specific miRNA inhibitor exhibits the potential to reinstate the therapeutic efficacy for IVDD both in vitro and in vivo.

**Conclusions:**

Taken together, our findings shed light on the mechanism of miRNAs sorting into sEVs and offer new insights for the optimization of sEV‐based treatments during intervertebral disc regeneration. regeneration.

## Graphical abstract

MiRNAs are selectively packed into MSC‐derived sEVs upon cellular stress

HnRNPU mediates the specific miRNAs sorting into sEVs and it enables the pro‐degenerative effect of sEVs

SNAP25 phosphorylation activates the nucleocytoplasmic transport of hnRNPU and facilitates the release of sEVs.

Specific miRNA inhibition alters the effect of oxidative‐stressed sEVs on intervertebral disc degeneration

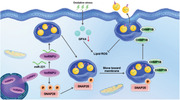

## 1 INTRODUCTION

Intervertebral disc degeneration (IVDD) is intricately associated with neck and back pain, thereby imposing a substantial burden on the worldwide healthcare system.^1^ The pathophysiology of IVDD is complicated and includes resident cell death, extracellular matrix (ECM) deterioration, inflammatory cytokine release and secondary changes in disc height and motility.^2^, ^3^ Amongst these, loss of nucleus pulposus (NP) cells as well as changes of their metabolic profile is the primary phenotype involved in IVDD.^4^ Exploring treatments aimed at restoring the functionality of viable NP cells is regarded as an essential realm of research within the field of intervertebral disc regeneration. Recently, regeneration approaches based on mesenchymal stem cells (MSCs) have shown promise in the NP cell therapy and restoring disc homeostasis.^5^ Understanding the underlying the mechanism of the MSC therapy could optimise the therapeutic efficiency and help to design novel approaches for disc regeneration.

Extracellular vesicles (EVs) are membrane‐like particles and have been identified as an important part of the MSC secretome.^6^ According to the International Society of Extracellular Vesicles, EVs can be categorised into small EVs (sEVs, 50−200 nm in diameter) and large EVs (> 200 nm) based on the physical size. Growing evidence has shown that sEVs derived from MSCs transfer functional miRNAs or proteins to recipient cells and manipulate the gene expression profile in the recipient cells.^7^, ^8^ Small EVs, including exosomes that originates from the endosomal membrane, have emerged as the cell‐free alternative of MSCs during the stem cell therapy. Our previous studies have focussed on the utilisation of sEVs in treating IVDD.^9^, ^10^, ^11^ Despite making some progress, our understanding of the mechanisms underlying EVs’ therapy remains incomplete. It is of great significance to acknowledge that the cellular state of MSCs influences the composition and functionality of the released sEVs.

Cellular stressors influence both the cellular status and the function, as well as the secretion of sEVs.^12^, ^13^ Numerous studies have highlighted the increased release of EVs in response to various stressful conditions, such as pressure overload, oxidative stress, acidity and hypoxia.^14^, ^15^, ^16^ Not only the quantity, but more importantly, the cargo contents of sEVs are greatly influenced by cell stressors. Especially, oxidative stress promotes the selective sorting of miRNAs into EVs that it improves the EVs function in immunoregulation.^12^ During this process, RNA‐binding proteins, such as heterogeneous nuclear ribonucleoprotein (hnRNP) mediate the specific loading of miRNAs into EVs.^17^ Our previous study has revealed that oxidative stress induces the ferroptosis of NP cells during IVDD, resulting in a decrease in protectors, such as ferroportin (FPN) and glutathione peroxidase 4 (GPX4).^18^ Herein, it is likely that the transplanted MSCs exposed to the oxidative stress environment in degenerated discs may alter the quantity and quality of their EV secretome.^19^, ^20^, ^21^ Investigating the changes in EV secretome under stressful conditions and elucidating the mechanisms involved in EV cargo sorting will provide new opportunities for the EV‐based therapy.

Ferroptosis is a type of cell death that is characterised by increased levels of lipid peroxidation and intracellular iron.^22^, ^23^ The recent study has demonstrated that ferroptosis is involved in promoting the formation of multivesicular bodies (MVBs) and the release of EVs.^24^ This serves as a protective mechanism that cells export EVs to decrease the intracellular iron ion level, resulting in a resistance to ferroptosis. Besides the iron exporting, the membrane repair mechanism is also involved in ferroptosis resistance.^25^ The endosomal sorting complexes required for transport (ESCRT) machinery plays a role in membrane repair and ameliorates the membrane damage caused by ferroptosis.^26^ Moreover, the ESCRT machinery participates in the secretion of EVs, and depletion of ESCRTs inhibits the production of EVs.^27^, ^28^, ^29^ It is reasonable to assume that cells may recruit ESCRT proteins to regulate EVs secretion and repair membrane damages upon the oxidative stress. Unravelling these mechanisms of EVs biogenesis and secretion could deepen our understanding of the MSC paracrine therapy and optimise the treatment efficiency.

In this study, we have found that oxidative stressed MSCs release a larger amount of sEVs. Moreover, we have observed that these oxidative sEVs have the ability to induce catabolic metabolism in NP cells, leading to the diminishing of the ECM. Subsequent analysis of the miRNA contents within the sEVs has revealed the enrichment of specific miRNAs. As a result, we hypothesise that these enriched miRNAs may mediate the effects of sEVs on NP cells. Through a screening of ESCRT proteins, we have determined the importance of charged multivesicular body protein 1B (CHMP1B) and synaptosomal‐associated protein 25 (SNAP25) in the biogenesis and secretion of sEVs. Additionally, we have identified the potential regulatory role of hnRNPU in selectively packing miRNAs into sEVs. Based on these findings, we have developed modified sEVs with miRNA‐221inhibitor and assessed their effects on IVDD. This study enhances the comprehension of the biogenesis and miRNA sorting mechanism of sEVs and introduces a new optimised approach for EV‐based intervertebral disc regeneration.

## 2 MATERIALS AND METHODS

### 2.1 Materials

Erastin and RSL‐3 were acquired from Selleck Chemicals (TX, USA). Ferrostatin‐1, Deferoxamine and Leptomycin B were purchased from MedChem Express (Shanghai, China). Phalloidin and tert‐butyl hydroperoxide (TBHP) were acquired from Sigma–Aldrich (MO, USA). All antibodies employed in the experiments were documented in Table S1.

### 2.2 Cell culture and coculture

Human NP cells and bone marrow‐derived MSCs were obtained from human tissues and cultured in Dulbecco's Modified Eagle Medium/Nutrient Mixture F‐12 as previously described.^10^ The culture medium contained 15% fetal bovine serum (ScienCell, CA, USA) and was refreshed every 3 days. For cell coculture, MSCs were cultured in a transwell insert (Corning, NY, USA) and subsequently cocultured with NP cells seeded in the lower chamber at 37°C under 5% CO_2_ and 20% O_2_. The number of MSCs remained equivalent to that of NP cells.

See Supporting information text for detailed materials and methods.

### 2.3 Statistical analysis

The data were presented as the mean ± standard deviation (SD) and all experiments were performed independently at least in triplicate. Student's *t*‐test was employed for comparing two groups. For multiple group comparisons, one‐way or two‐way analysis of variance (ANOVA) with Tukey's post hoc test was utilised. Statistical significance was measured using GraphPad Prism 8 software (La Jolla, CA, USA) and a *P*‐value < .05 denoted a statistical difference (*P < .05, **P < .01, ***P < .001).

## 3 RESULTS

### 3.1 Oxidative stress induces the ferroptosis of mesenchymal stem cells and alters their paracrine effects

**FIGURE 1:**
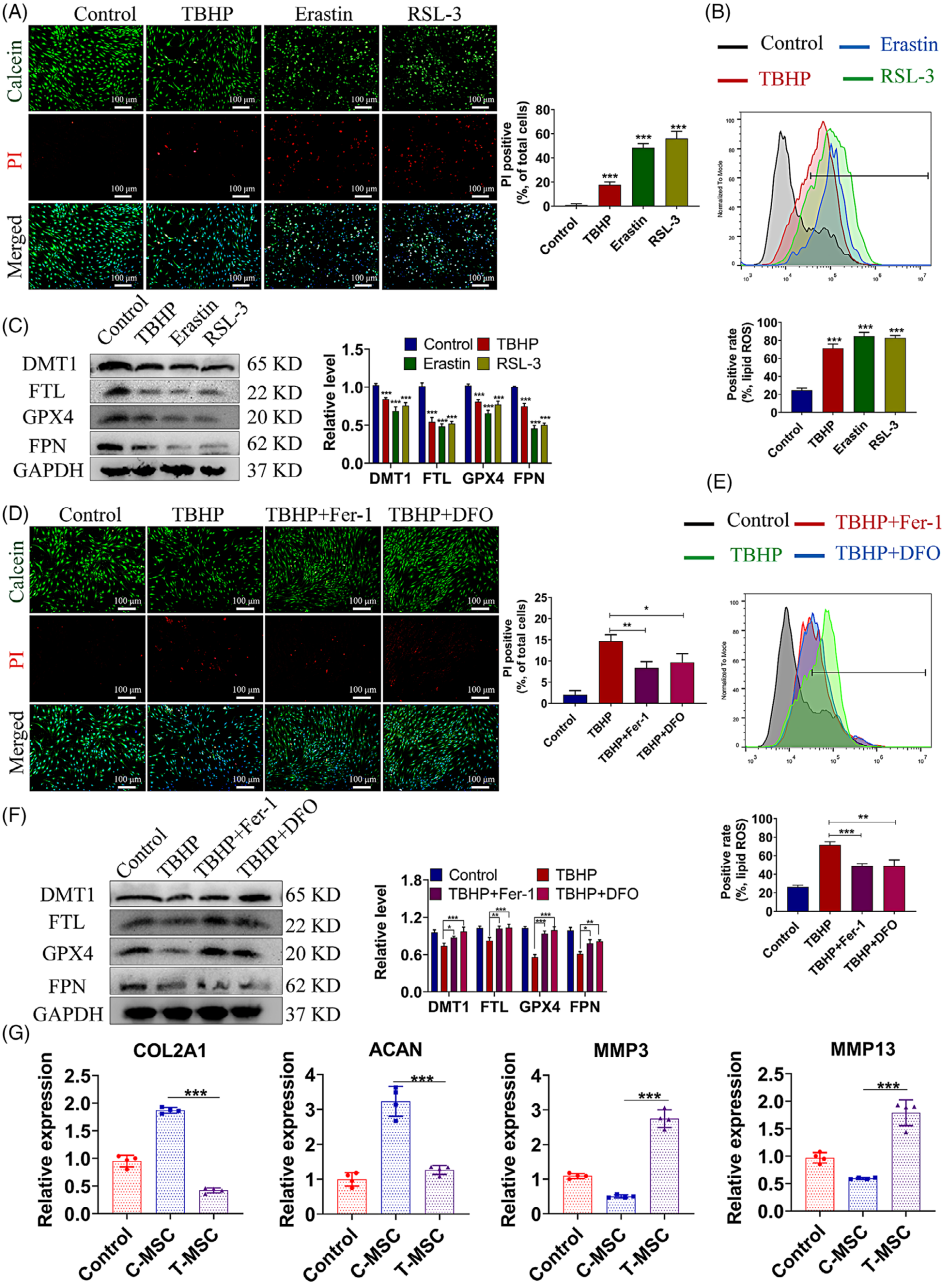
Oxidative stress promotes MSCs ferroptosis and affects the coculture outcome with NP cells. (A–C) MSCs were treated with TBHP, Erastin, or RSL‐3 for 12 h. (A) Live/dead staining images and the quantitative Propidium iodide (PI) positive rate of total cells. (B) Flow cytometry of C11‐BODIPY 581/591 and the quantitative positive rate indicating the lipid ROS level. (C) Western blot analysis of DMT1, FTL, GPX4, and FPN in MSCs and the quantitative relative protein levels. (D–F) MSCs were treated with TBHP, ferrostatin‐1 (Fer‐1), or deferoxamine (DFO) for 12 h. (D) Live/dead staining images and the quantitative PI positive rate. (E) Flow cytometry of C11‐BODIPY 581/591 and the quantitative lipid ROS level. (F) Western blot analysis of DMT1, FTL, GPX4, and FPN in MSCs and the quantitative relative protein levels. (G) MSCs were pre‐treated with TBHP (50 μm) for 12 h (T‐MSC) or common medium (C‐MSC). T‐MSCs or C‐MSCs were then cocultured with NP cells for 24 h. The mRNA levels of COL2A1, ACAN, MMP3, and MMP13 in NP cells were measured by RTqPCR. NP cells without MSCs coculture were used as control. Data were presented as mean ± SD of at least three independent replicates. *P < .05, **P < .01 and ***P < .001. MSC, mesenchymal stem cell; NP, nucleus pulposus; TBHP, tert‐butyl hydroperoxide; SD, standard deviation.

TBHP as a common reagent was used to induce oxidative stress. We initially found that TBHP induced the decrease of MSCs viability and promoted the death rate in a dose‐dependent manner (Figure S1A–C). It was indicated that the TBHP treatment elicited the accumulation of lipid Reactive oxygen species (ROS) (Figure S1D). Besides, we also found that the TBHP treatment increased the cell death rate and lipid ROS level in a time‐dependent manner (Figure S2A–D). We then chose a moderate concentration and time duration of the TBHP treatment (50 μm, 12 h) in the following experiments. For further assessments, we compared the effects of TBHP with two known ferroptosis inducers Erastin and RSL3, on the death of MSCs and the level of lipid ROS (Figure 1A,B). Similar to Erastin and RSL3, the TBHP treatment induced the decreased expression of DMT1, FTL, FPN and GPX4 proteins, implying the overload of iron and ferroptosis (Figure 1C). However, the effect of TBHP on cell viability and lipid ROS level was ameliorated when the ferroptosis inhibitors were used (Figure 1D,E). The levels of ferroptosis‐associated proteins were also increased by the co‐treatments of inhibitors in TBHP‐treated MSCs (Figure 1F). Importantly, based on our previous coculture model,^10^ we found that the paracrine effects of TBHP‐treated MSCs (T‐MSC) on NP cells differed from the normal MSCs (C‐MSC). Specifically, T‐MSC coculture induced the decreased expression of COL2A1 (type II collagen) and ACAN (aggrecan), and the increased expression of MMP3 and MMP13 (matrix metalloproteinase, MMP3 and 13) (Figure 1G). Our findings indicated that the co‐culture of T‐MSCs induced the catabolic metabolism of NP cells, thereby highlighting the detrimental impact of T‐MSC paracrines on IVDD.

### 3.2 Specific miRNAs are selectively packed into small extracellular vesicles upon oxidative stress

**FIGURE 2:**
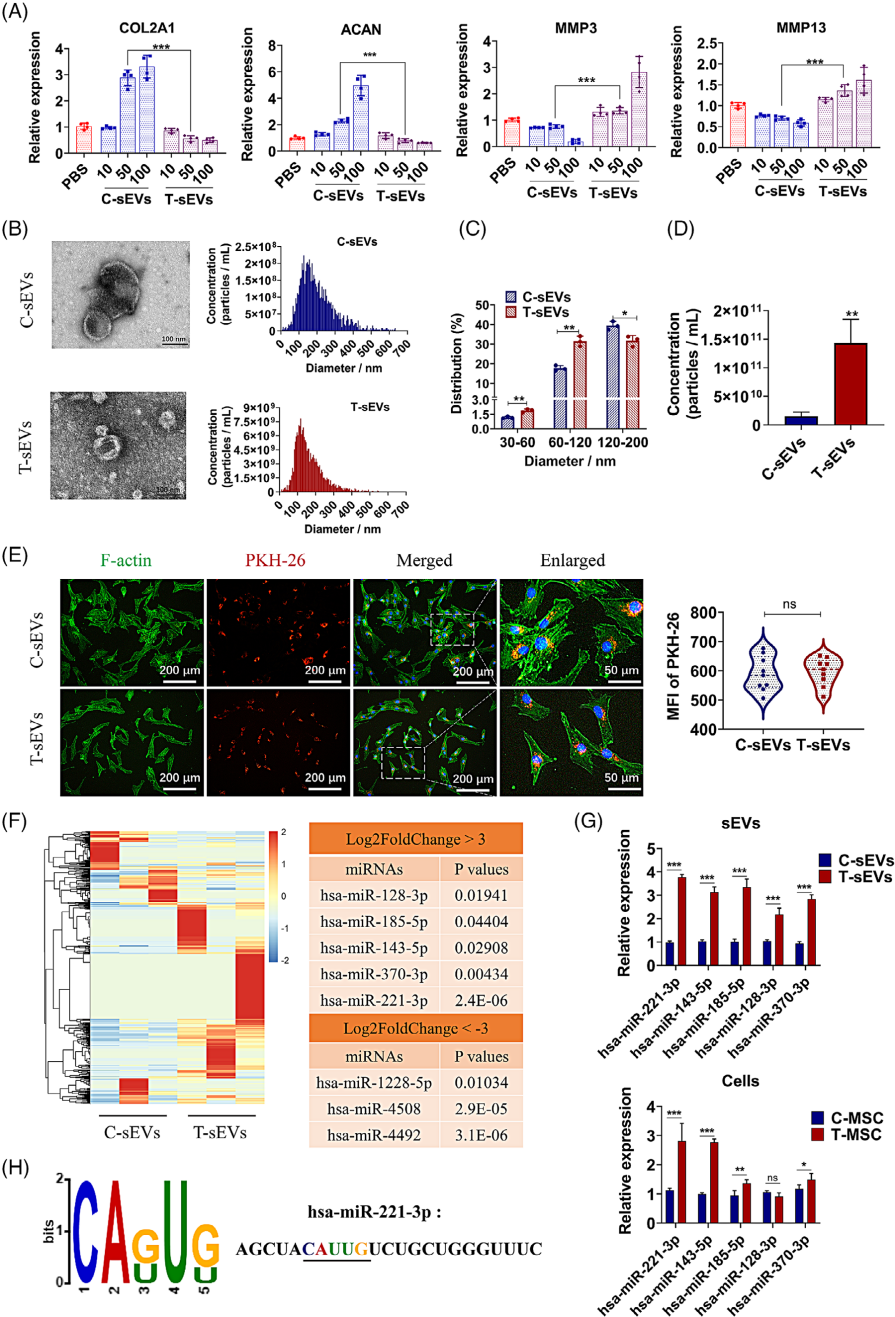
Oxidative stress alters the miRNA expression in sEVs and the sEVs‐mediated effects on NP cells. (A) The sEVs were isolated from control MSCs (C‐sEVs) or TBHP‐treated MSCs (T‐sEVs) and incubated with NP cells. The mRNA levels of COL2A1, ACAN, MMP3 and MMP13 in NP cells were measured (10, 50, 100 μg/mL of sEVs). (B) Representative transmission electron microscopy images and nanoparticle tracking analysis (NTA) of sEVs. (C,D) Particle diameter distribution (C) and concentration (D) of C‐sEVs and T‐sEVs measured by NTA. (E) Images of PKH‐26‐labelled sEVs (red) internalised by NP cells and the quantitative mean fluorescence intensity (MFI) of PKH‐26. Phalloidin (green) for F‐actin and DAPI (blue) for nuclei. (F) Heatmap of differential expressed miRNAs in T‐sEVs vs. C‐sEVs, and the representative miRNAs with fold changes over ± 3. (G) The levels of five up‐regulated miRNAs in T‐sEVs were measured in sEVs fraction (above) and in cell fraction (below). (H) Enriched motif of up‐regulated miRNAs in T‐sEVs and the base sequence of has‐miR‐221‐3p (most significant). Data were presented as mean ± SD of at least three independent replicates. *P < .05, **P < .01, ***P < .001, and ns for no significant difference. sEV, small extracellular vesicle; MSC, mesenchymal stem cell; NP, nucleus pulposus.

To further evaluate the effects of MSC paracrine, the sEVs were isolated from C‐MSC (C‐sEVs) and T‐MSC (T‐sEVs) and then incubated with NP cells. We observed that T‐sEVs decreased the levels of COL2A1 and ACAN, and increased the levels of MMP3 and MMP13 in NP cells (Figure 2A). The size and morphology of these EVs were in accord with the definition of sEVs (Figure 2B). Of note, the proportion of small EVs with diameter less than 120 nm was higher in T‐sEVs than in C‐sEVs (Figure 2C). The concentration of T‐sEVs was much higher and almost ten times of C‐sEVs (Figure 2D). On the other hand, the uptake rates of C‐sEVs and T‐sEVs by NP cells were without significant difference (Figure 2E). We further analysed the miRNAs in C‐sEVs and T‐sEVs. We observed a total of 26 up‐regulated and 30 down‐regulated miRNAs in T‐sEVs (Figure S3A). Enrichment analysis of these differentially expressed miRNAs indicated many ECM associated biological process and signalling pathways (Figure S3B–D). Especially, miR‐128/185/143/370/221 were notably up‐regulated (more than three‐folds) in T‐sEVs and miR‐221 was with the most minimum *P‐*value (Figure 2F). We then measured the expression levels of these five miRNAs and found that miR‐221 was up‐regulated significantly both in parental MSCs and sEVs samples (Figure 2G). Further motif analysis of all 26 up‐regulated miRNAs in T‐sEVs detected a conserved CAUUG motif (Figure 2H). About 60% of up‐regulated miRNAs in T‐sEVs contained this motif, but over 90% of up‐regulated miRNAs in C‐sEVs lacked this motif. Besides, the motif combined match *P*‐value of miR‐221 was the most significant amongst the up‐regulated miRNAs in T‐sEVs. In all, our results indicated that certain miRNAs were preferentially encapsulated into sEVs in TBHP‐treated MSCs.

### 3.3 SNAP25 plays an important role in the biogenesis of small extracellular vesicles in oxidative stressed mesenchymal stem cells

**FIGURE 3:**
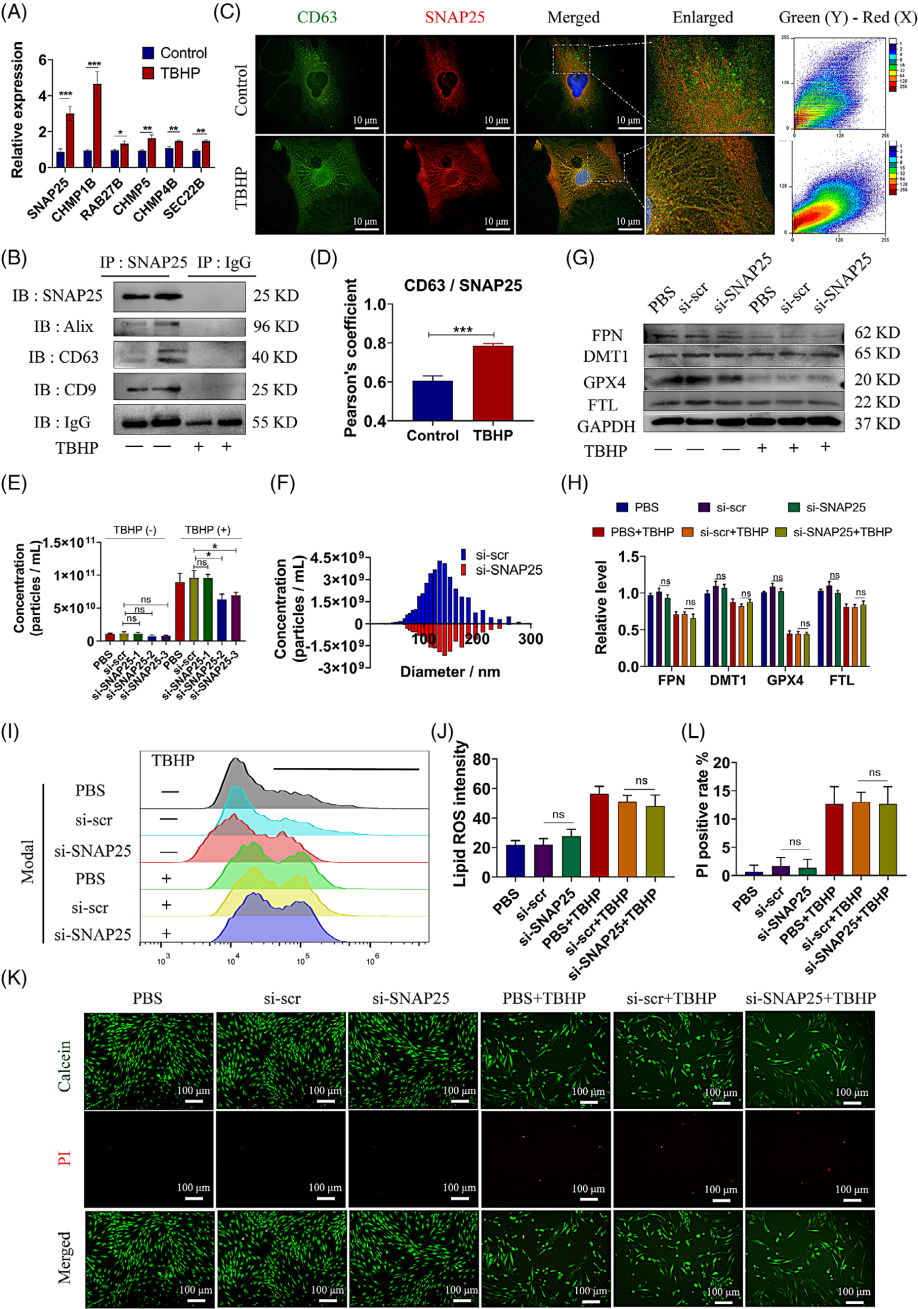
SNAP25 affects the sEVs release in MSCs. (A) The mRNA levels of six up‐regulated genes in TBHPtreated MSCs were measured. (B) Immunoprecipitation of SNAP25 and immunoblotting of SNAP25, Alix, CD63 and CD9 in TBHP‐treated and control MSCs. IgG was used as a negative control. (C,D) Immunofluorescent images of SNAP25 (red) and CD63 (green) in TBHP‐treated and control MSCs (C) and distribution of fluorescence intensity in the enlarged part (right panel). The colocalisation Pearson coefficient was calculated (D). (E,F) Particle concentration of sEVs derived from MSCs transfected with three types of si‐SNAP25 or scrambled siRNA (si‐scr) was measured (E), and the distribution of particle diameter (F, partial data). (G–L) Phosphate buffered saline (PBS) or TBHP‐treated MSCs were transfected with or without siRNAs (si‐SNAP25 or si‐scr). Western blot images of DMT1, FTL, GPX4, and FPN in MSCs (G) and the quantitative relative protein levels (H). Flow cytometry of C11‐BODIPY 581/591 in MSCs (I) and the quantitative lipid ROS levels (J). Live/dead staining images of MSCs (K) and the quantitative PI positive rate (L). Data were presented as mean ± SD of at least three independent replicates. *P < .05, **P < .01, ***P < .001, and ns for no significant difference. sEV, small extracellular vesicle; MSC, mesenchymal stem cell; SNAP25, synaptosomal‐associated protein 25; TBHP, tert‐butyl hydroperoxide.

In order to reveal the mechanism of sEVs biogenesis and release in oxidative stressed MSCs, we conducted a transcriptome sequencing in MSCs. It was indicated that there were 955 up‐regulated and 1 119 down‐regulated genes in TBHP‐treated MSCs compared with the control group (Figure S4A,B). Gene Ontology (GO) and pathway analysis showed that up‐regulated genes were related to oxidative stress or stimulus response (Figure S4C,D). We further screened some molecules which may be involved in the biogenesis of sEVs (Figure S5A). It was revealed that the expression levels of SNAP25 and CHMP1B were with remarkable significance (Figure 3A). We further observed that SNAP25 significantly bound to sEVs‐related tetraspanin proteins in TBHP‐treated MSCs, including CD63, CD9 and Alix (Figure 3B). Besides, it was also indicated the colocalisation of SNAP25 and CD63 in MSCs (Figure 3C,D). The expression levels of SNAP25 were decreased after the specific siRNAs transfection in MSCs (Figure S5B,C). It was observed that the secretion of sEVs was markedly reduced in TBHP‐treated MSCs when si‐SNAP25 was employed, resulting in an approximate decrease of 30% in sEVs production (Figure 3E,F). Besides, the expression levels of ferroptosis‐related proteins were not significantly altered in SNAP25 down‐regulated MSCs (Figure 3G,H). The lipid ROS levels and cell viability in si‐SNAP25‐treated MSCs were also similar to the control group (Figure 3I–L). It was shown that SNAP25 played a critical role in regulating the secretion of sEVs in MSCs, while exerting minimal influence on ferroptosis susceptibility.

### 3.4 CHMP1B‐mediated membrane repair facilitates small extracellular vesicles release and protects against cell ferroptosis

**FIGURE 4:**
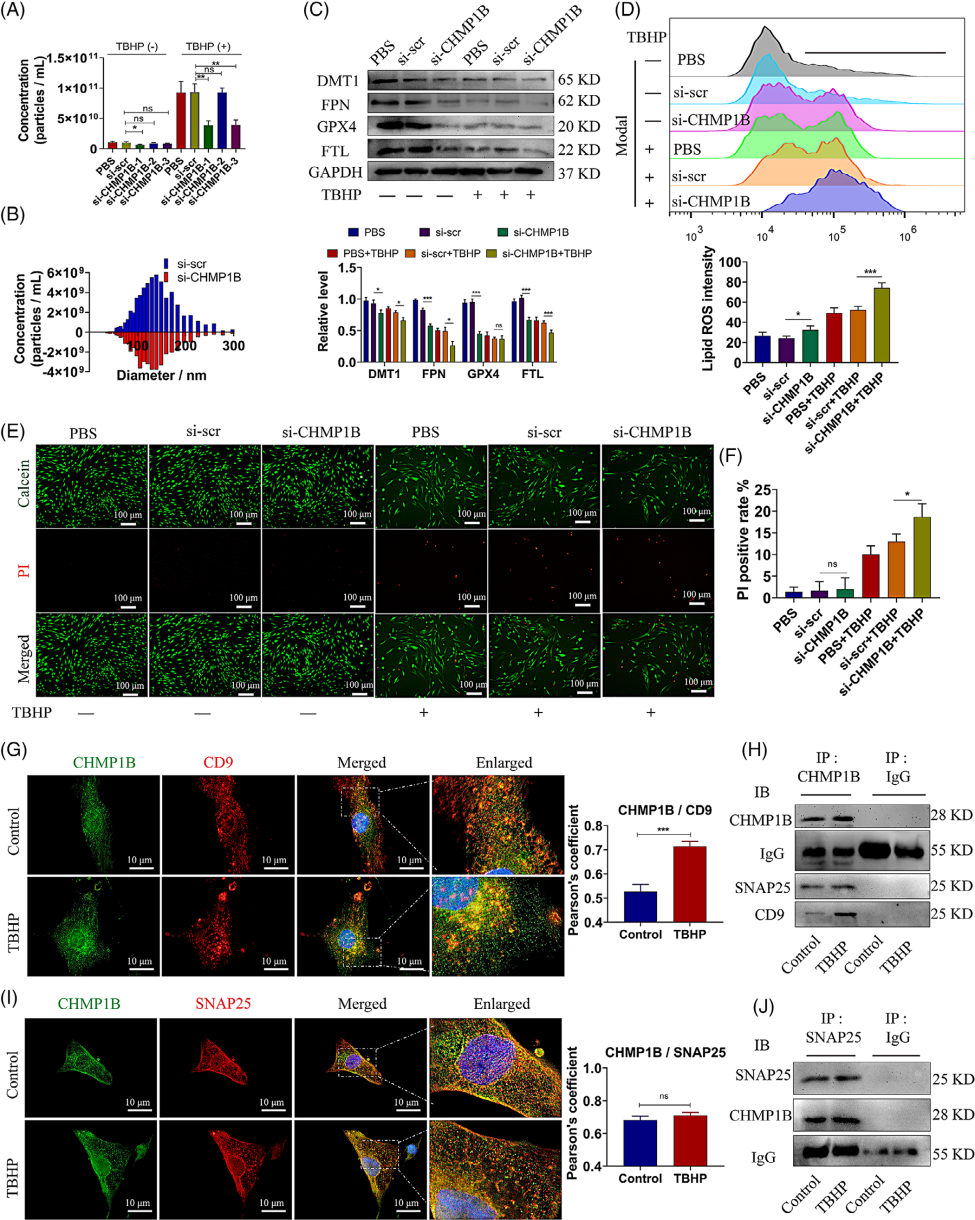
CHMP1B regulates the sEVs release in MSCs. (A) Particle concentration of sEVs derived from MSCs transfected with three types of si‐CHMP1B or scrambled siRNA (si‐scr) was measured, and the distribution of particle diameter (B, partial data). (C–F) PBS or TBHP‐treated MSCs were transfected with or without siRNAs (si‐CHMP1B or si‐scr). Western blot images of DMT1, FTL, GPX4 and FPN in MSCs (C) and the quantitative relative protein levels. Flow cytometry of C11‐BODIPY 581/591 in MSCs (D) and the quantitative lipid ROS levels. Live/dead staining images of MSCs (E) and the quantitative PI positive rate (F). (G–J) MSCs were treated with PBS (control) or TBHP. (G) Immunofluorescent images of CHMP1B (green) and CD9 (red), and the Pearson coefficient was calculated. (H) Immunoprecipitation of CHMP1B and immunoblotting of CHMP1B, SNAP25 and CD9. (I) Immunofluorescent images of CHMP1B (green) and SNAP25 (red), and the Pearson coefficient were calculated. (J) Immunoprecipitation of SNAP25 and immunoblotting of SNAP25 and CHMP1B.IgG was used as a negative control. Data were presented as mean ± SD of at least three independent replicates. *P < .05, **P < .01, ***P < .001, and ns for no significant difference. sEV, small extracellular vesicle; MSC, mesenchymal stem cell; SNAP25, synaptosomal‐associated protein 25; TBHP, tert‐butyl hydroperoxide; CHMP1B, charged multivesicular body protein 1B.

We utilised siRNAs to interfere the expression of CHMP1B in MSCs (Figure S5D,E). Knockdown of CHMP1B in MSCs caused a reduction in the release of sEVs, with an approximately 60% decrease in production compared to the control group (Figure 4A,B). We also found that CHMP1B knockdown reduced the expression of ferroptosis‐related proteins, accompanied by the increased lipid ROS level and cell death rate (Figure 4C–F). It was discovered that knockdown of CHMP1B exacerbated the injury on cells. Thus, we hypothesised that CHMP1B was implicated in the mechanism of membrane repair and fusion. To test this hypothesis, we detected the interaction between CHMP1B and both SNAP25 and CD9. We observed that the colocalisation of CHMP1B and CD9 was significant in TBHP‐treated MSCs (Figure 4G). Furthermore, there was an elevation in the level of CHMP1B/CD9 complex in MSCs treated with TBHP, as depicted in Figure 4H. We also evaluated the integration and colocalisation between CHMP1B and SNAP25, which indicated the potential existence of CHMP1B/SNAP25/CD9 protein complex (Figure 4I,J). In summary, our findings demonstrated that CHMP1B was involved in the secretion of sEVs through the formation of a protein complex, and it also provided protection against ferroptosis in MSCs.

### 3.5 Nucleocytoplasmic transport of hnRNPU regulates specific miRNAs sorting into small extracellular vesicles

**FIGURE 5:**
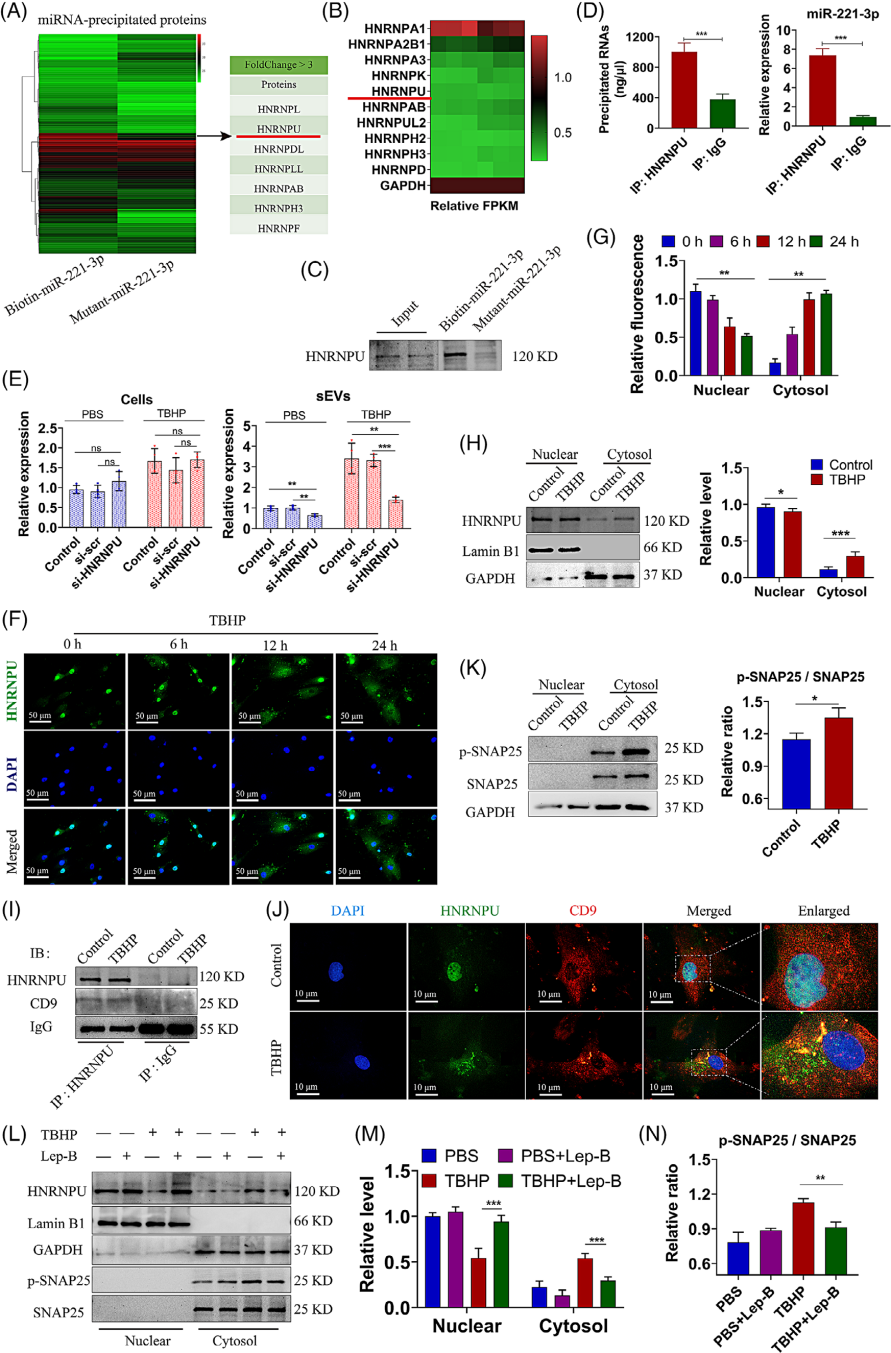
HNRNPU regulates the miRNAs sorting of sEVs. (A) Heatmap of miRNA‐precipitated proteins, and the representative differential expressed HNRNPs with fold changes over three. (B) Heatmap of high abundance HNRNPs in TBHP‐treated MSCs vs. normal MSCs. (C) Western blot images of HNRNPU pull‐down by miR‐221‐3p or mutant‐miR‐221‐3p in MSCs. (D) RIP analysis of total RNA concentration and the relative level of miR‐221‐3p precipitated by HNRNPU or IgG was measured. IgG was used as a negative control. (E) PBS or TBHP‐treated MSCs were transfected with si‐HNRNPU or si‐scr. The level of miR‐221‐3p in cells fraction or sEVs fraction was measured. (F) Immunofluorescent images of HNRNPU (green) in MSCs treated with TBHP (0, 6, 12, 24 h), and the mean fluorescence intensity of HNRNPU in nuclear and cytosol (G). (H) Nuclear and cytoplasmic HNRNPU expression in control or TBHP‐treated MSCs were measured by western blot analysis, and the relative protein level was calculated. Lamin B1 or GAPDH served as loading control of the nucleus or cytoplasm, respectively. (I) Immunoprecipitation of HNRNPU and immunoblotting of HNRNPU and CD9 in TBHP‐treated and control MSCs. (J) Immunofluorescent images of HNRNPU (green) and CD9 (red) in TBHP‐treated and control MSCs. (K) Western blot images of phosphorylated SNAP25 (p‐SNAP25) and SNAP25 in control or TBHP‐treated MSCs, and the ratio of p‐SNAP25/SNAP25 was calculated. (L‐N) PBS or TBHP‐treated MSCs were co‐treated with leptomycin B. (L) Western blot images of HNRNPU, p‐SNAP25 and SNAP25. (M) The relative protein level of nuclear and cytoplasmic HNRNPU was calculated. (N) The ratio of p‐SNAP25/SNAP25 was calculated. Data were presented as mean ± SD of at least three independent replicates. *P < .05, **P < .01, ***P < .001, and ns for no significant difference. sEV, small extracellular vesicle; MSC, mesenchymal stem cell; SNAP25, synaptosomal‐associated protein 25; TBHP, tert‐butyl hydroperoxide.

We designed a mutant biotinylated miR‐221 sequence without the CAUUG motif which served as the control. Then, we utilised these biotinylated miRNAs to pull down the RNA‐binding proteins (Figure S6A). Some hnRNPs were significantly up‐regulated in biotinylated miR‐221 precipitates (Figure 5A). The previous transcriptome sequencing identified some hnRNPs that displayed high expression levels in both control and TBHP‐treated MSCs (Figure 5B). Based on the findings depicted in Figure 5A,B, hnRNPU emerged as the most plausible candidate for miR‐221 binding. Furthermore, hnRNPU was detected in the miR‐221 precipitates not the mutant miR‐221 precipitates, indicating that specific binding of hnRNPU and miR‐221 in MSCs (Figure 5C). Through the immunoprecipitation of hnRNPU, we observed a significant upregulation of miR‐221, with an approximately 7‐fold increase compared to the IgG negative control (Figure 5D). Other differential expressed miRNAs such as miR‐143, miR‐185, miR‐128 and miR‐370 were detected in hnRNPU immunoprecipitates. It was found that miRNAs with CAUUG motif were with elevated expression relative to IgG negative control (Figure S6B). In addition, we also assessed the expression of several miRNAs (*P* > .05) that were enriched in hnRNPU immunoprecipitates. Our findings indicated that these miRNAs exhibited no differential expression compared to the IgG negative control (Figure S6C), and some miRNAs exhibited low affinity with hnRNPU (CT > 40). These results provided support for the notion that miRNAs with specific motifs were more likely to be packaged into sEVs with the assistance of hnRNPU.

In the further experiments, we used siRNAs to knockdown the expression of hnRNPU (Figure S6D). We observed that the expression level of miR‐221 was not altered by hnRNPU knockdown in MSCs but significantly reduced in MSC‐derived sEVs (Figure 5E). When focussing on the distribution of hnRNPU, an observation was made that the TBHP treatment caused a time‐dependent increase in cytosolic hnRNPU levels (Figure 5F,G). Further analysis of protein levels also indicated a decrease in the nuclear hnRNPU level but an increase in the cytosolic hnRNPU level in TBHP‐treated MSCs (Figure 5H). Additionally, further investigation revealed an integration and colocalisation between cytosolic hnRNPU and CD9 (Figure 5I,J). Our findings suggested that TBHP induced both the translocation of hnRNPU and the phosphorylation of SNAP25 in MSCs (Figure 5K). Furthermore, the inhibition of cytosolic translocation of hnRNPU by leptomycin B significantly hindered the phosphorylation of SNAP25 (Figure 5L–N). Therefore, these results provided evidence that hnRNPU selectively packaged miR‐221 into sEVs and participated in the biogenesis of sEVs through the translocation of hnRNPU between the nucleus and cytosol.

### 3.6 MiR‐221 inhibition allows small extracellular vesicles to recover the therapeutic effect on nucleus pulposus cells

**FIGURE 6:**
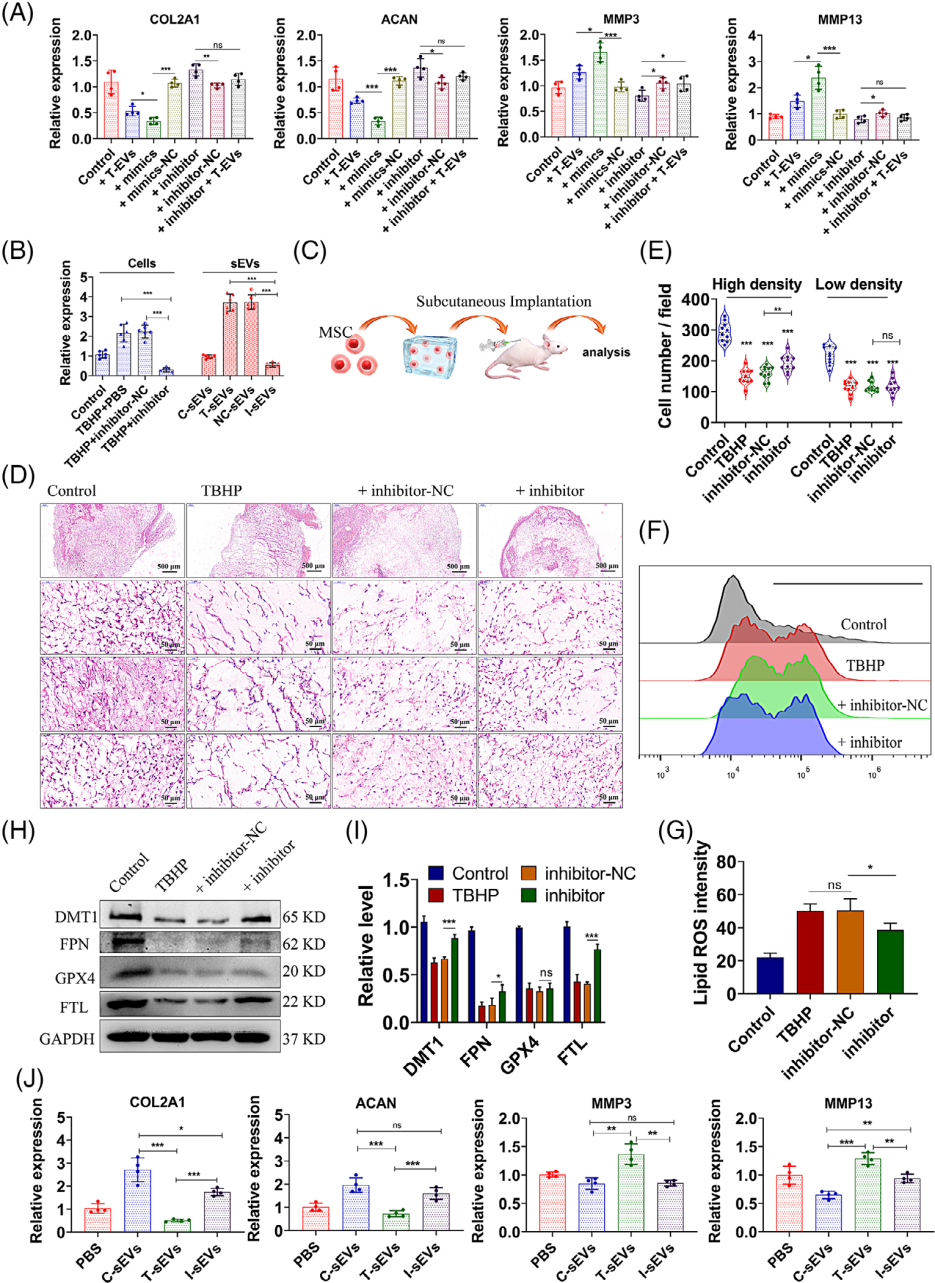
MiR‐221 inhibition alters the effects of sEVs on NP cells in vitro. (A) NP cells were transfected with miR‐221 mimics, mimics control (mimics‐NC), inhibitors or inhibitor control (inhibitor‐NC), or incubated with sEVs derived from TBHP‐treated MSCs (T‐sEVs). The mRNA levels of ACAN, MMP3 and MMP13 in NP cells were measured. (B) MSCs co‐treated with TBHP were transfected with miR‐221 inhibitor or inhibitors‐NC and the sEVs were isolated (I‐sEVs and NC‐sEVs, respectively). The C‐sEV was derived from control MSCs treated with PBS. The levels of miR‐221‐3p in C‐sEVs, T‐sEVs, NC‐sEVs and I‐sEVs, as well as their parent MSCs were measured. (C) Schematic graph of MSCs subcutaneous implantation model. MSCs were pretreated with TBHP and transfected with miR‐221 inhibitor or inhibitor‐NC, and then implanted in the nude mice. (D,E) Representative histological staining images of the implanted cell block (D), and the quantitative results of cell numbers (E). The count fields with relatively high or low density were randomly selected. (F–I) PBS or TBHP‐treated MSCs were transfected with miR‐221 inhibitor or inhibitor‐NC. Flow cytometry of C11‐BODIPY 581/591 (F) and the quantitative lipid ROS levels (G). Western blot analysis of DMT1, FTL, GPX4, and FPN in MSCs (H) and the quantitative relative protein levels (I). (J) The NP cells were incubated with equivalent C‐sEVs, T‐sEVs, I‐sEVs or PBS. The mRNA levels of ACAN, MMP3, and MMP13 in NP cells were measured. Data were presented as mean ± SD of at least three independent replicates. *P < .05, **P < .01, ***P < .001, and ns for no significant difference. sEV, small extracellular vesicle; MSC, mesenchymal stem cell; NP, nucleus pulposus; TBHP, tert‐butyl hydroperoxide.

After transfected with miR‐221 mimics or inhibitors, the levels of miR‐221 in NP cells were evaluated (Figure S7A). We observed that both miR‐221 mimics transfection and T‐sEVs treatment resulted in the upregulation of MMP3 and MMP13 expression, as well as the downregulation of COL2A1 and ACAN expression (Figure 6A). However, MiR‐221 inhibitor significantly abrogated the effects of T‐sEVs. Besides, we utilised mimics and inhibitors of other differential expressed miRNAs (miR‐143, miR‐185, miR‐128 and miR‐370) to treat NP cells. Our observations revealed that miR‐143, miR‐185 and miR‐128, which possessed similar sequences as miR‐221, also exerted effects on ECM metabolism. Conversely, miR‐370, lacking the CAUUG motif, exhibited no significant alteration in the expression of COL2A1 and MMP3 (Figure S7B). These enlightening results indicated that the decreased level of miR‐221 may eliminate the adverse effect of TBHP‐treated sEVs, and miR‐221 may be one of the culprits for adverse effects. We then attempted to knockdown the miR‐221 level in MSCs via a miR‐221 inhibitor and isolated the sEVs (I‐sEVs) fraction. The expression level of miR‐221 in cells and sEVs fraction was measured in TBHP‐treated MSCs with the transfection of miR‐221 inhibitor or negative NC control (Figure 6B). The protein levels of Alix, CD63 and CD9 were measured (Figure S7C), indicating equal utilisation of sEVs across different groups. Importantly, miR‐221 expression in I‐sEVs exhibited a statistically significant reduction when compared to T‐sEVs.

To further investigate the effects of miR‐221, we utilised a subcutaneous implantation model to evaluate the cell viability in vivo (Figure 6C). Pre‐treatment with TBHP was employed to induce oxidative stress prior to cell implantation. We observed that viability of oxidative stressed MSCs significantly decreased compared with the control group (Figure 6D). However, MSCs transfected with miR‐221 inhibitor seemed to obtain a higher cell number compared with the negative NC control (Figure 6E). Moreover, our results revealed that miR‐221 knockdown could decrease the ROS level in TBHP‐treated MSCs (Figure 6F,G). The expression levels of ferroptosis‐related proteins were found to be increased in miR‐221 inhibitor‐transfected MSCs (Figure 6H,I). It was also revealed that less shrunken mitochondria were detected in miR‐221 inhibitor‐transfected MSCs (Figure S7D). These findings demonstrated that the suppression of miR‐221 enhanced the survival of MSCs under conditions of oxidative stress. In this case, we evaluated the effects of sEVs from modified MSCs on NP cells, and found that I‐sEVs significantly induced the decreased expression of MMP3 and MMP13, and the increased expression of COL2A1 and ACAN compared with T‐sEVs (Figure 6J). It was indicated that the inhibition of miR‐221 partially restored the beneficial effects of sEVs on NP cells.

### 3.7 Comparative effects of small extracellular vesicles with different miRNAs ingredient on disc degeneration in vivo

**FIGURE 7:**
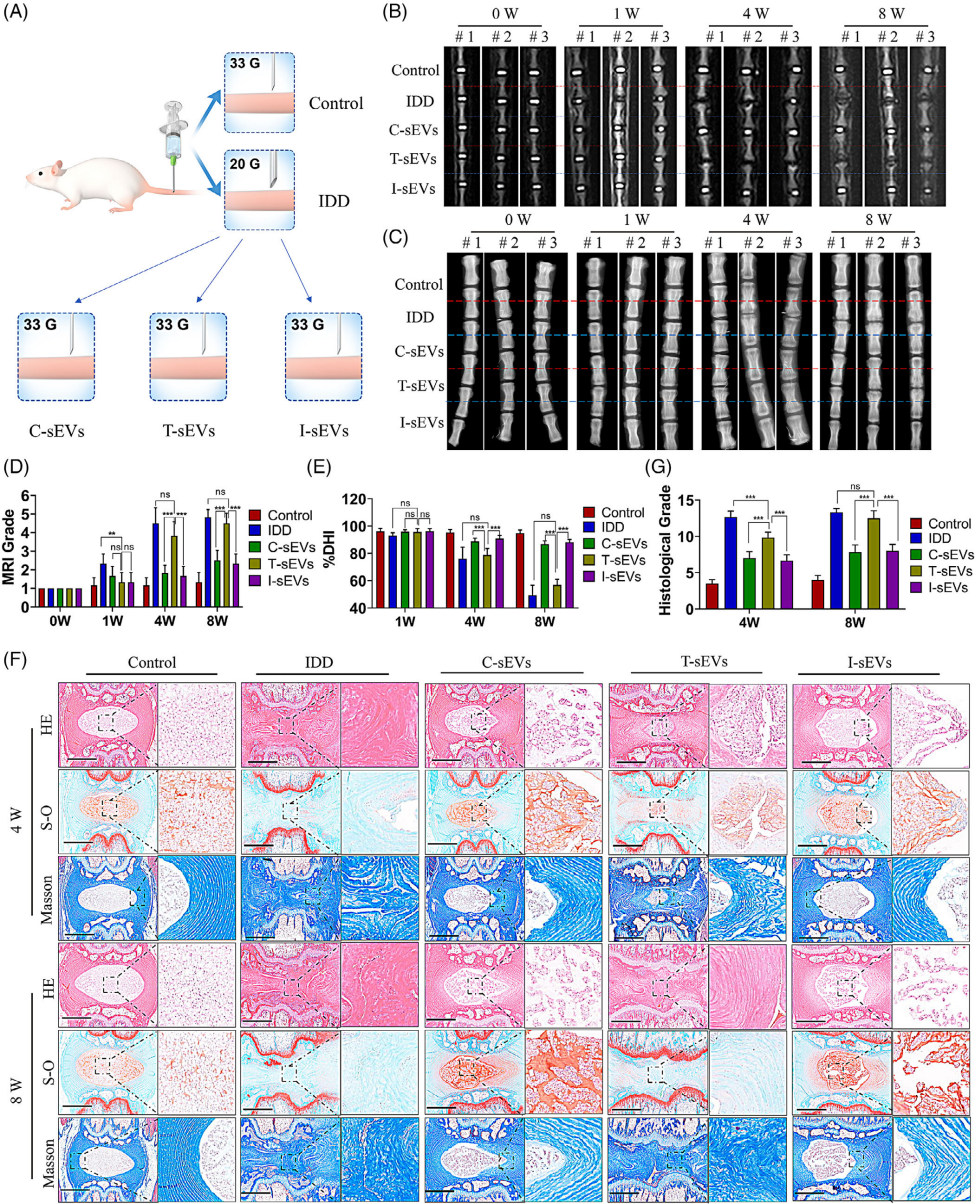
MiR‐221 inhibition alters the effects of sEVs on IDD in vivo. (A) Schematic graph of disc degeneration and sEVs therapy model. The 20 G needle was used to initiate disc degeneration and 33 G needle was for sEVs injection. Three types of sEVs were used, including C‐sEVs (from control MSCs), T‐sEVs (from TBHP‐treated MSCs), and I‐sEVs (from MSCs transfected with miR‐221 inhibitor). (B) Representative MRI images of discs treated with different sEVs in different segments at 0, 1, 4 and 8 weeks. Three random individuals were shown. (B) Representative X‐ray images of discs treated with different sEVs in different segments at 0, 1, 4 and 8 weeks. Three random individuals were shown. (D) Quantitative results of MRI grade based on the MRI images. (E) Quantitative results of DHI% based on the X‐ray images. (F) Hematoxylin‐eosin (HE), Safranin‐O (S‐O), and Masson staining images of discs treated with different sEVs at 4 and 8 weeks. (G) Quantitative results of histological grade based on the histological staining images. Data were presented as mean ± SD of at least three independent replicates. **P < .01, ***P < .001, and ns for no significant difference. sEV, small extracellular vesicle; MSC, mesenchymal stem cell; TBHP, tert‐butyl hydroperoxide.

To further evaluate the effects of sEVs, we utilised a rat disc degeneration model for sEVs delivery (Figure 7A). The degenerated disc is characterised by a loss of water content and collagens, a narrow disc space, and a decreased NP area.^30^ We observed that the Magnetic resonance imaging (MRI) grade in the C‐sEVs and I‐sEVs group was lower compared with the T‐sEVs group, suggesting a higher moisture content within the intervertebral disc (Figure 7B,D). The Disc height index (DHI%) in the C‐sEVs and I‐sEVs group was with smaller drop compared with the Intervertebral disc degeneration (IDD) and T‐sEVs group, indicating the disc space with no obvious collapse (Figure 7C,E). These results demonstrated that both C‐sEVs and I‐sEVs could ameliorate the IVDD, whereas T‐sEVs accelerate the progression of IVDD. Further histological analysis showed that the discs in the C‐sEVs and I‐sEVs group were less degenerated compared with the T‐sEVs group. This was evidenced by the greater presence of inner NP areas, a higher density of NP cells, and a lower histological grade according to the histological staining (Figure 7F,G). Besides, the level of aggrecan in both the C‐sEVs and I‐sEVs groups was higher compared to the T‐sEVs group (Figure S8A,B). Conversely, the level of MMP3 in the C‐sEVs and I‐sEVs groups was lower than in the T‐sEVs group (Figure S8C,D). Taken together, these findings indicated that sEVs derived from oxidative‐stressed MSCs were not suitable for the treatment of IVDD. However, inhibiting miR‐221 in donor MSCs could restore the therapeutic effect of their sEVs.

## 4 DISCUSSION

**FIGURE 8:**
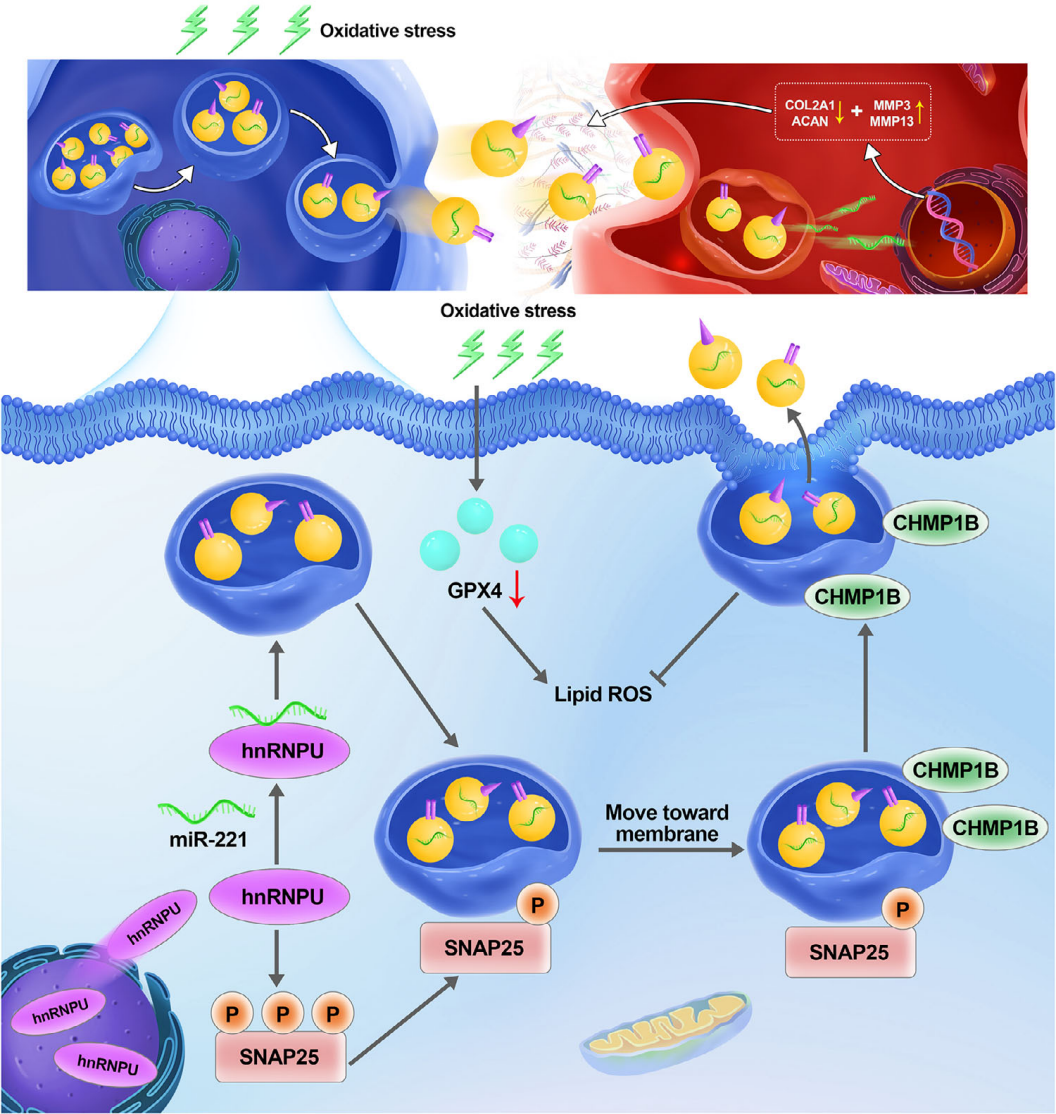
Proposed model of the selective sorting of specific miRNAs into oxidative stressed MSCs. Under oxidative stress, the nucleus‐cytosol translocation of HNRNPU facilitates the sorting of miR‐221 into intraluminal vesicles. Additionally, HNRNPU mediates the phosphorylation of SNAP25, promoting the transport of vesicles towards the membrane. Membrane repair mediated by CHMP1B not only protects against cell ferroptosis but also contributes to the fusion of intraluminal vesicles with the cytomembrane, subsequently leading to the release of sEVs. Importantly, sEVs derived from oxidatively stressed MSCs possess the ability to promote the catabolic metabolism of NP cells. sEV, small extracellular vesicle; MSC, mesenchymal stem cell; NP, nucleus pulposus; SNAP25, synaptosomal‐associated protein 25; CHMP1B, charged multivesicular body protein 1B.

In this study, we found that oxidative stress could induce the ferroptosis of MSCs and subsequently the abundant release of sEVs. Besides, the selected miRNAs were sorted into sEVs due to the change of MSCs state. These oxidative sEVs could educate NP cells a catabolic metabolism, mainly via the delivery of miR‐221. The present data also showed that SNAP25 and CHMP1B were involved in the biogenesis and secretion of sEVs. CHMP1B‐mediated membrane repairing was also the protective mechanism to cell ferroptosis. Moreover, hnRNPU regulated the selective packing of miR‐221 into sEVs and mediated the phosphorylation of SNAP25 via its nuclear export. These results unravelled the molecular mechanism of sEVs secretion in oxidative stressed MSCs and the function of sEVs in recipient NP cells (Figure 8). We also found that the sEVs with minimal miR‐221 could regain the therapeutic effects on NP cells in vitro and in vivo. It provides novel insight into how to optimise the application of MSC–sEVs during disc regeneration.

MSCs have been utilised in the therapeutic management of diverse diseases, encompassing musculoskeletal disorders, neurodegenerative diseases, shock, and tumors.^31^, ^32^, ^33^ MSCs are commonly employed for the repair of tissues or organs through cell transplantation.^34^ They serve as scaffolds to provide viable cells or bioactive molecules. They also can serve as immune regulators by generating and secreting cytokines for the anti‐tumour therapy.^31^ Currently, MSCs have been used for intervertebral disc regeneration, mainly through cell differentiation or their paracrine effects.^35^ The hallmark of IVDD is the degradation of ECM components and the infiltration of fibrosus tissues. During the progression of IVDD, NP cells are inclined to catabolic metabolism with a high expression of MMPs and the decreased collagens.^36^ The exogenous MSCs are capable to differentiate into NP‐like cells, which produce and maintain the ECM.^37^ Besides, paracrine secretion from MSCs could induce the ECM synthesis of local NP cells and reduce cell apoptosis.^38^, ^39^ EVs are the important ingredient of MSCs paracrine secretion. EVs are the potential cell‐free therapy which serves as an alternative of MSCs.^40^ Compared to the MSCs therapy, EVs are the ideal choice for tissue regeneration, which are convenient in preservation, transportation and administration.^41^ Our previous studies have focussed on the application of MSC‐EVs in treating IVDD.^9^, ^10^ However, there are multiple factors that affect the EVs therapeutic effect.

Many studies have shown that MSC‐derived EVs play a role in NP cells matrix remodelling.^42^, ^43^ Xing et al. developed a new hydrogel based on EVs delivery mainly promote a healthier ECM production in treating IVDD.^43^ Our study also reveals the therapeutic effects of common MSC‐derived sEVs on degenerated NP cells. However, the quantity and quality of secreted EVs are affected by the state of parental MSCs.^9^ Our findings suggest that MSCs subjected to oxidative stress exhibit alterations in the composition of their paracrine sEVs. The sEVs released by oxidative MSCs induce a catabolic phenotype in NP cells, thus impairing the expected therapeutic effect. These findings support the hypothesis that sEVs derived from MSCs residing in an oxidative microenvironment may have detrimental effects on the treatment of IVDD. Hence, it is imperative to explore the underlying mechanism of sEVs biogenesis, as it could offer valuable insights into the development of EVs‐based therapeutic strategies.

EVs are once considered as the trash emerging in the process of cytomembrane repairing.^44^ Recent studies have shown that the secretion of EVs could serve as the regulation mechanism of cellular homeostasis.^24^, ^45^ Brown et al. found that ferritin selectively packed into MVBs and release outside via small EVs to protect against cell ferroptosis.^24^ Besides, the previous study has indicated that EVs could pack and export cellular mitochondrial proteins to prevent the cytoplasmic accumulation of mitochondrial damage‐associated molecular patterns.^45^ It appears that the secretion of EVs serves as a significant cellular compensatory mechanism during periods of stress. Nevertheless, it is noteworthy that the composition of EVs, including their enclosed RNAs and proteins, may undergo concurrent alterations.

Ferroptosis is a type of oxidative stress‐induced regulated cell death, which is mainly driven by lipid peroxidation and iron accumulation.^46^ Dai et al. found that ESCRT‐mediated membrane repair is an important machinery to resist ferroptosis, promoting cell survival under oxidative stress.^47^ Our study indicated that CHMP1B regulates the release of sEVs. The blockage of CHMP1B significantly decreased the secretion of sEVs. Many studies have shown that ESCRTs are activated to repair membrane or promote endolysosomal repair to antagonise cell death.^48^, ^49^, ^50^ Our findings are in line with previous studies. In addition, our data revealed that downregulation of SNAP25 leads to a significant decrease in the release of sEVs, while having minimal impact on the susceptibility to ferroptosis. Conversely, CHMP1B is associated with the process of membrane repair, and knockdown of CHMP1B exacerbates cell ferroptosis. Based on these observations, it is plausible to conclude that the fusion of MVBs and subsequent release of EVs represents the mechanism behind ESCRT‐mediated membrane repair.

EVs communicate with recipient cells via the delivery of functional RNAs, proteins or lipids.^8^ MicroRNAs are abundant in small EVs and considered as the main functional substance.^51^, ^52^ Our findings demonstrate the enrichment of specific miRNAs in sEVs derived from MSCs treated with TBHP. Notably, miR‐221 exhibits a pronounced pro‐catabolic effect. Based on our observations, it is plausible to hypothesise that oxidative stress triggers the production of miR‐221, and its subsequent release via sEVs may serve as a regulatory mechanism employed by MSCs. Importantly, the inhibition of miR‐221 in sEVs significantly abrogates its pro‐catabolic effect, thus highlighting the ability of parental MSCs to modify their paracrine effects. Indeed, the sorting of miRNAs in sEVs is selectively and the underlying mechanism is not fully understood.^53^ Many studies have reported the role of HNRNP proteins in the sorting of miRNAs.^12^, ^54^, ^55^, ^56^ Lee et al. found that hnRNPA2B1 mediates the sorting of miR‐198 into microvesicles under noxious stimuli.^12^ The previous study has shown that hnRNPU plays a role in the process of miR‐30c packaging into large EVs.^56^ In our present investigation, it was observed that miRNAs containing the CAGUG motif exhibit a higher likelihood of being enriched by hnRNPU, in contrast to miRNAs harboring a mutated CAGUG motif that did not precipitate with hnRNPU. These findings suggest a potential role for hnRNPU in mediating the selective sorting of specific miRNAs into sEVs in oxidatively stressed MSCs, consequently influencing the effects of sEVs on recipient cells.

Engineered EVs are constructed by strategies that focussed on the design of EVs cargo or membrane molecule.^57^ So far, EVs are considered as the effective way for nucleic acid delivery.^58^ One study has designed a CD9‐HuR membrane functionalised EVs to realise the enrichment of specific miRNA.^59^ Liang et al. utilised miRNA inhibitor oligonucleotide‐loaded EVs to achieve a significant anti‐tumour effect.^60^ In the present study, we have discovered that miR‐221 is highly enriched in sEVs derived from oxidative stressed MSCs, and this particular miRNA is responsible for mediating the pro‐catabolic effect of sEVs. To mitigate this detrimental effect during the EVs therapy, we have utilised a miR‐221 inhibitor to treat the parental MSCs before isolating the modified sEVs. Subsequently, in further investigations, we have observed that these modified sEVs, exhibiting significantly decreased expression of miR‐221, have the ability to facilitate the anabolic metabolism of degenerated NP cells and impede IVDD in a rat model. It should be noted that the presence of oxidative stress risk factors in the microenvironment during MSC application may potentially influence the paracrine release of EVs. Our study supports the notion that the adverse effects of sEVs can be averted by the thoughtful design of parental MSCs.

To further illustrate the role of sEVs in MSC‐based disc regeneration, several issues still remain to be investigated in the future. First, we used TBHP to simulate a microenvironment of oxidative stress and focussed on the change of sEVs miRNA cargoes. Actually, the degenerated disc microenvironment is complicated and harsh, that includes oxidative stress, inflammation, acidity and pressure. Other contents, such proteins and lipids may also play a role in the communication between MSCs and NP cells. Second, multiple proteins may be involved in the miRNA sorting into sEVs. Our study only investigates the role of hnRNPU based on the binding assay of miR‐221. Third, our study needs to investigate the modification of RNA‐binding proteins or miRNAs. To unravel the modification mechanism could deepen the understanding of the sorting process and may provide potential targets for the sEVs therapy.

## 5 CONCLUSION

In summary, our study elucidates the mechanism underlying the sorting of miRNAs into EVs in response to oxidative stress. This discovery highlights the impact of the cellular state on EV secretion, offering insights into the therapeutic applications of MSC paracrine. Furthermore, we have identified a key miRNA responsible for mediating catabolic effects on NP cells. Genetic manipulation to suppress this miRNA has shown the potential to restore the therapeutic properties of sEVs. These findings pave the way for further investigation into the potential use of MSC‐derived sEVs in the treatment of IVDD.

## AUTHOR CONTRIBUTIONS

*Conceptualization, Experimental operation, Data collection, Methodology, Writing manuscript*: Zhiwei Liao. *Conceptualization, Experimental operation*: Bide Tong. *Conceptualization, Experimental operation*: Xiaoguang Zhang. *Data collection*: Weifeng Zhang. *Data collection*: Wencan Ke.*Experimental operation*: Huaizhen Liang. *Data collection*: Ming Lei. *Methodology*: Wenbin Hua. *Methodology*: Shuai Li. *Conceptualization, Data curation, Supervision*: Yu Song. *Conceptualization, Data curation, Funding acquisition, Supervision*: Xinghuo Wu. *Conceptualization, Data curation, Funding acquisition, Supervision, Writing manuscript*: Cao Yang.

## CONFLICT OF INTEREST STATEMENT

The authors declare no conflicts of interest.

## ETHICAL APPROVAL

All the experimental protocols were approved by the Ethics Committee of Tongji Medical College, Huazhong University of Science and Technology (Nos. 174).

## Supporting information

Supporting Information

## Data Availability

The data that support the findings of this study are available from the corresponding author upon reasonable request.
