# Supplementary material for: Selective cargo sorting in stem cell‐derived small extracellular vesicles: impact on therapeutic efficacy for intervertebral disc degeneration
Source: Clin Transl Med. 2023 Nov 30;13(12):e1494. doi: 10.1002/ctm2.1494 (PMC10689973; doi:10.1002/ctm2.1494)
Supplement: Supplementary file 1 — Supporting Information [file CTM2-13-e1494-s001.docx]

**Supplemental Information**

**Selective Cargo Sorting in Stem Cell-Derived Small Extracellular Vesicles: Impact on Therapeutic Efficacy for Intervertebral Disc Degeneration**

Zhiwei Liao**^#^**, Bide Tong**^#^**, Xiaoguang Zhang**^#^**, Weifeng Zhang, Wencan Ke, Huaizhen Liang, Ming Lei, Wenbin Hua, Shuai Li, Yu Song*****, Xinghuo Wu*****, and Cao Yang*****

Department of Orthopaedics, Union Hospital, Tongji Medical College, Huazhong University of Science and Technology, Wuhan 430022, China

**^#^** These authors contributed equally to this work

***** To whom correspondence should be addressed:

Cao Yang, Department of Orthopaedics, Union Hospital, Tongji Medical College, Huazhong University of Science and Technology, Wuhan 430022, China

E-mail address: caoyangunion@hust.edu.cn

Xinghuo Wu, Department of Orthopaedics, Union Hospital, Tongji Medical College, Huazhong University of Science and Technology, Wuhan 430022, China

E-mail address: wuxinghuo@163.com

Yu Song, Department of Orthopaedics, Union Hospital, Tongji Medical College, Huazhong University of Science and Technology, Wuhan 430022, China

E-mail address: songyumail@hust.edu.cn

**Materials and Methods**

**Isolation of sEVs**

MSCs were cultured in 15% fetal bovine serum (VivaCell, Shanghai, China) depleted of sEVs. The sEVs were isolated using a differential centrifugation method. In brief, the culture medium was subjected to consecutive centrifugation steps at 500 g for 10 minutes, 2000 g for 30 minutes, and 12000 g for 1 hour. The supernatant was filtered using a 0.22-μm filter (Millipore, MA, USA) and subsequently subjected to 110000 g centrifugation (Beckman Type 70 Ti, CA, USA) for 70 min twice. The resulting pellet represented the sEVs fraction, which was then resuspended in PBS.

**Transmission electron microscopy**

For transmission electron microscopy (TEM) analysis, the sEVs samples were fixed with 2.5% glutaraldehyde for a duration of 30 minutes and subsequently mounted on copper grids. In addition, the cell samples were fixed, embedded in an epoxy resin, and sectioned into ultra-thin slices. Subsequently, random views of all the samples were captured utilizing a TEM instrument (FEI Tecnai, OR, USA).

**Nanoparticle tracking analysis**

For nanoparticle tracking analysis (NTA), the sEVs were diluted and tracked by NANOSIGHT NS300 system (Malvern, UK) configured with a 488 nm laser and a high-sensitivity sCMOS camera. The diameter distribution and concentration of particles was measured by the NTA software (Malvern, UK).

**Labelling of sEVs and internalization Assay**

The sEVs were labeled using a red fluorescent labeling reagent, PKH26 (5 μM, Sigma-Aldrich, MO, USA), followed by washing with PBS and centrifugation at 110000 g for 70 min to remove any excess dye. Subsequently, the labeled sEVs were incubated with NP cells at 37℃ for 12 h. After staining the cytoskeleton using phalloidin (Beyotime, Shanghai, China), the samples were observed under a fluorescence microscope (Olympus, Japan). Randomly selected target views were used for analysis, and the mean fluorescence intensity (MFI) was calculated using ImageJ 1.5 (National Institutes of Health, MD, USA).

**Cell viability assay**

The cell viability of NP cells was evaluated using the Cell Counting Kit-8 (CCK-8, Beyotime, Shanghai, China). An equivalent number of cells were seeded in a 96-well plate and subsequently exposed to the experimental interventions. Afterward, a 10% CCK-8 solution was added and incubated for a duration of 4 hours. The absorbance of the samples at 450 nm was measured using a spectrophotometer (BioTek, VT, USA).

**Living and dead staining**

Living and dead cells were stained using the Calcein/PI kit (Beyotime, Shanghai, China). Calcein-AM was employed for staining viable cells, while propidium iodide (PI) was applied to stain dead cells. The cells were incubated with the Calcein/PI solution for 30 minutes in the absence of light. After being washed twice in PBS, the samples were observed under a fluorescence microscope (Olympus, Japan), and images were captured at random views. The number of PI-positive cells was subsequently quantified using ImageJ 1.5 software.

**Western blot analysis**

The cellular proteins were extracted using the RIPA solution (Beyotime, Shanghai, China). The nuclear and cytoplasmic proteins were isolated using Nuclear and Cytoplasmic Protein Extraction Kit (Beyotime, Shanghai, China). The samples were separated by 12.5% sodium dodecyl sulfate polyacrylamide gel electrophoresis in tris-glycine running buffer at a voltage of 110V. Subsequently, the proteins were transferred onto a PVDF band (Millipore, USA). The membrane was washed in blocking buffer (NCM Biotech, Nanjing, China) for 15 min at room temperature, followed by overnight incubation with a primary antibody. After washing, the band was incubated with a horseradish peroxidase (HRP)-conjugated secondary antibody and visualized using an enhanced chemiluminescence reagent (Thermo Fisher, MA, USA). Images were captured using a Bio-Rad system (ChemiDoc MP, USA). The density of bands in three independent experiments was quantified using ImageJ 1.5 software, and the levels were normalized to GAPDH.

**Lipid ROS analysis**

Lipid ROS levels were assessed using the C11-BODIPY 581/591 (Thermo Fisher, MA, USA) fluorescent dyes. In brief, cells were treated with 10 μM C11-BODIPY 581/591 and incubated at 37°C for 30 minutes. Subsequently, the cells were washed twice, trypsinized, and resuspended in PBS. The samples were then analyzed using a FACSCalibur flow cytometer (BD Biosciences, NJ, USA), with lipid peroxidation fluorescence shifts analyzed using FlowJo X software (Tree Star, MA, USA). The excitation wavelength was set at 488 nm and the emission wavelength was measured at 525/530 nm, indicating the production of lipid ROS.

**Immunofluorescence analysis**

Cells were fixed with 4% paraformaldehyde for 30 min and permeabilized with 0.2% Triton X-100 for 15 min. Following this, the samples were blocked with 2% goat serum albumin for an hour and then incubated with primary antibodies overnight. After washing with PBS, the samples were exposed to fluorescent-conjugated secondary antibodies for an hour under dark conditions. Nuclei were stained by DAPI (Beyotime, Shanghai, China) for 5 min, and images were captured using a microscope (Olympus, Japan) or a confocal microscope (Nikon, Japan) by three independent researchers. The colocalization analysis was conducted using ImageJ 1.5 software by three independent researchers.

**Quantitative real-time PCR (RT-qPCR)**

Total cellular RNA was extracted using TRIzol (Invitrogen, CA, USA) and subjected to chloroform separation. The purity and concentration of RNA were assessed using a Nanodrop-1000 spectrophotometer (Thermo Fisher, MA, USA). Subsequently, the purified RNA was reverse transcribed utilizing a cDNA synthesis kit and further subjected to RT-qPCR using the SYBR Green qPCR kit (Vazyme, Nanjing, China). U6 and β-actin served as internal controls. The specific primers utilized for RT-qPCR analysis can be found in supplemental Table S2.

**RNA transfection**

Small interfering RNA (siRNA), scrambled siRNA (si-scr), miRNA mimics, miRNA inhibitors and the negative control (NC) were chemically synthesized and subsequently dissolved in diethyl pyrocarbonate (20 μM) for storage. The sequences have been provided in supplemental table S3. Cells were cultured in 24-well plate until 50% confluency and then transfected with siRNAs or miRNAs (100 nM) using PolyFast (MCE, Shanghai, China) according to manufacturer's instruction. The cell culture medium was replaced 6 hours after transfection, and the efficiency of interference was assessed at 24 hours post-transfection using RT-qPCR.

**Immunoprecipitation**

Protein immunoprecipitation was performed by extracting cellular proteins using a solution containing 50 mM Tris-HCl, 150 mM NaCl, 1 mM EDTA, and 1% NP-40, supplemented with a protease inhibitor cocktail (Beyotime, Shanghai, China). The sample was then mixed with a precipitation antibody or IgG (10 μg) and incubated overnight with magnetic beads (MCE, Shanghai, China). The immunoprecipitates were separated using magnetic adsorption and washed twice with PBS. The isolated immunoprecipitates were subsequently subjected to western blot assays. For RNA immunoprecipitation, proteins were treated with a solution containing 50 mM Tris-HCl, 150 mM NaCl, 1 mM EDTA, 1% NP-40, and 100 U/ml RNase inhibitor, supplemented with a protease inhibitor cocktail. The reagents involved in this process were prepared using diethyl pyrocarbonate. After incubation with the precipitation antibody, the precipitates were isolated and RNAs were extracted using TRIzol and chloroform. The precipitated RNAs were then subjected to RT-qPCR assay.

**RNA pull down**

MiRNAs were biotinylated and subsequently combined with cell lysates. Following treatment with 100 U/ml RNase inhibitor and protease inhibitor cocktail, the 5 nmol of biotinylated miRNAs were incubated with protein lysates at 4℃ for 6 hours. Pre-washed streptavidin magnetic beads (2 mg/mL, MCE, Shanghai, China) were added to the mixture and incubated at 4℃ for 2 hours. Subsequently, the mixture was isolated using magnetic force and washed twice with washing buffer. Finally, the sample was subjected to western blot analysis or mass spectrometry.

**RNA sequencing**

The miRNAs in sEVs were extracted using the exoRNeasy Midi Kit (Qiagen, Germany). The cellular RNAs were extracted using TRIzol (Invitrogen, CA, USA). The isolated miRNAs or mRNAs were utilized for the preparation of RNA sequencing libraries (Illumina, CA, USA). The obtained raw data was subjected to quantification and mapping against the mature miRNA sequence or mapping to the human genome using STRA 2.5 software. Differential expression analysis was conducted on RNAs between groups, considering a significance level of P < 0.05. Gene ontology (GO) analysis and pathway enrichment analysis for the differentially expressed genes were performed using the DAVID Functional Annotation Bioinformatics tool. Additionally, the analysis of miRNA motifs was carried out using the Multiple Em for Motif Elicitation tool (MEME).

**Subcutaneous implantation**

The BALB/c-nu nude mice (male, 8-week, 18g) were obtained from the Experimental Animal Center of Tongji Medical College, Huazhong University of Science and Technology. In order to assess the viability of MSCs, cell pellets (5×10^7^/ml) were seeded in alginate hydrogel (1%, wt%) and subsequently subcutaneously implanted at the back of the nude mice for 2 weeks. Following the implantation, the implanted pellets were collected, fixed in formaldehyde, and embedded in paraffin. The paraffin blocks were then sectioned into 4-μm slices and stained with hematoxylin and eosin using the Servicebio HE staining kit (Wuhan, China). The resulting images were captured, and the cell numbers were quantified by counting the cells under three randomly selected fields using ImageJ 1.5 software.

***In vivo* disc experiments**

Sprague-Dawley (male, 8-week, 200g) rats were purchased from the Experimental Animal Center of Tongji Medical College, Huazhong University of Science and Technology. A surgical model of IVDD was established using needle puncture. The intervertebral discs at levels Co 6/7, 7/8, 8/9, 9/10, and 10/11 were identified by palpation and confirmed by radiography. The Co 6/7 disc served as the sham group, punctured using a 33-gauge needle, while the Co 7/8 disc represented the IVDD group, punctured using a 20-gauge needle. The Co 8/9, 9/10, and 10/11 discs were punctured using a 20-gauge needle, and followed by injection of different types of sEVs (50 μg/mL, 2 μL) using a 33-gauge needle. The injections of sEVs were performed weekly and lasted for 8 weeks.

**Radiological imaging assay**

The discs were examined through radiography using an *in vivo* MS FX PRO imaging system (Bruker, USA). The disc height was measured and the disc height index (DHI) was calculated. The change in DHI was utilized to assess disc degeneration, and it was calculated using the formula: DHI % = post-DHI / pre-DHI × 100%. The post-DHI referred to the DHI after the surgical operation, while the pre-DHI represented the DHI before the operation. Magnetic resonance imaging (MRI) was performed using an MRI system (BRUKER BioSpec 7T, Germany), and sagittal T2-weighted images were used to assess the signal of the discs. Pfirrmann grades, based on T2-weighted MRI sections (Grade I indicating a normal disc and Grade V indicating severe disc degeneration), were employed to evaluate the disc degeneration.

**Histological analysis**

The rats were euthanized by asphyxiation, and subsequently, the discs were collected, fixed in formaldehyde, and decalcified using EDTA. Following this, the samples were dehydrated and embedded in paraffin. The paraffin blocks were then sectioned into 4-μm slices and stained with hematoxylin and eosin (HE), Safranin O-fast green (S-O), or Masson. The degenerative state of discs was evaluated based on a histological grading scale, which consisted of 5 categories of disc changes, assigning 0 points for a normal disc and 15 points for a severely degenerated disc.

For immunohistochemical analysis, the sections were deparaffinized, rehydrated, and subjected to microwave treatment using sodium citrate. Subsequently, the samples were blocked with 3% bovine serum albumin at room temperature for 30 min. The sections were then incubated with a primary antibody overnight, followed by three washes with PBS. After incubation with HRP-conjugated secondary antibody at room temperature for 1h, the sections were washed with PBS and then stained with hematoxylin for 3 min. The images were captured using a microscope (Olympus, Japan).

**Supplemental Figures (S1-S8)**


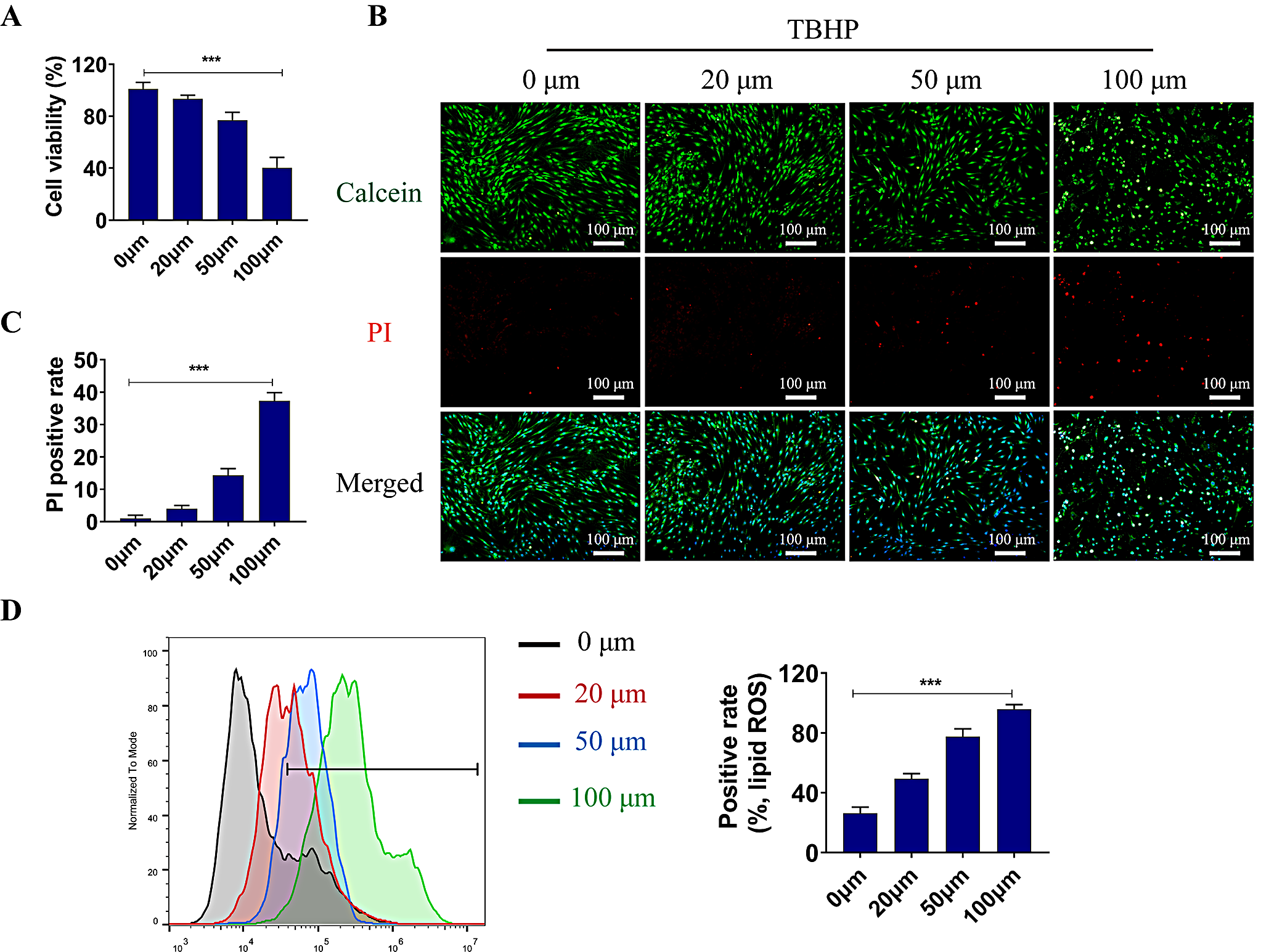


**Figure S1:** TBHP induces the ferroptosis of MSCs in a dose-dependent manner. MSCs were treated with different doses of TBHP (0 μm, 20 μm, 50 μm, 100 μm) for 12 h. (A) CCK-8 results of MSCs. (B-C) Live/dead staining of MSCs (B), and the quantitative PI positive rate of total MSCs (C). (D) Lipid ROS level of MSCs (left) and the corresponding quantitative results of lipid ROS level (right). Data were presented as mean ± SD of at least three independent replicates. ****P* < 0.001.


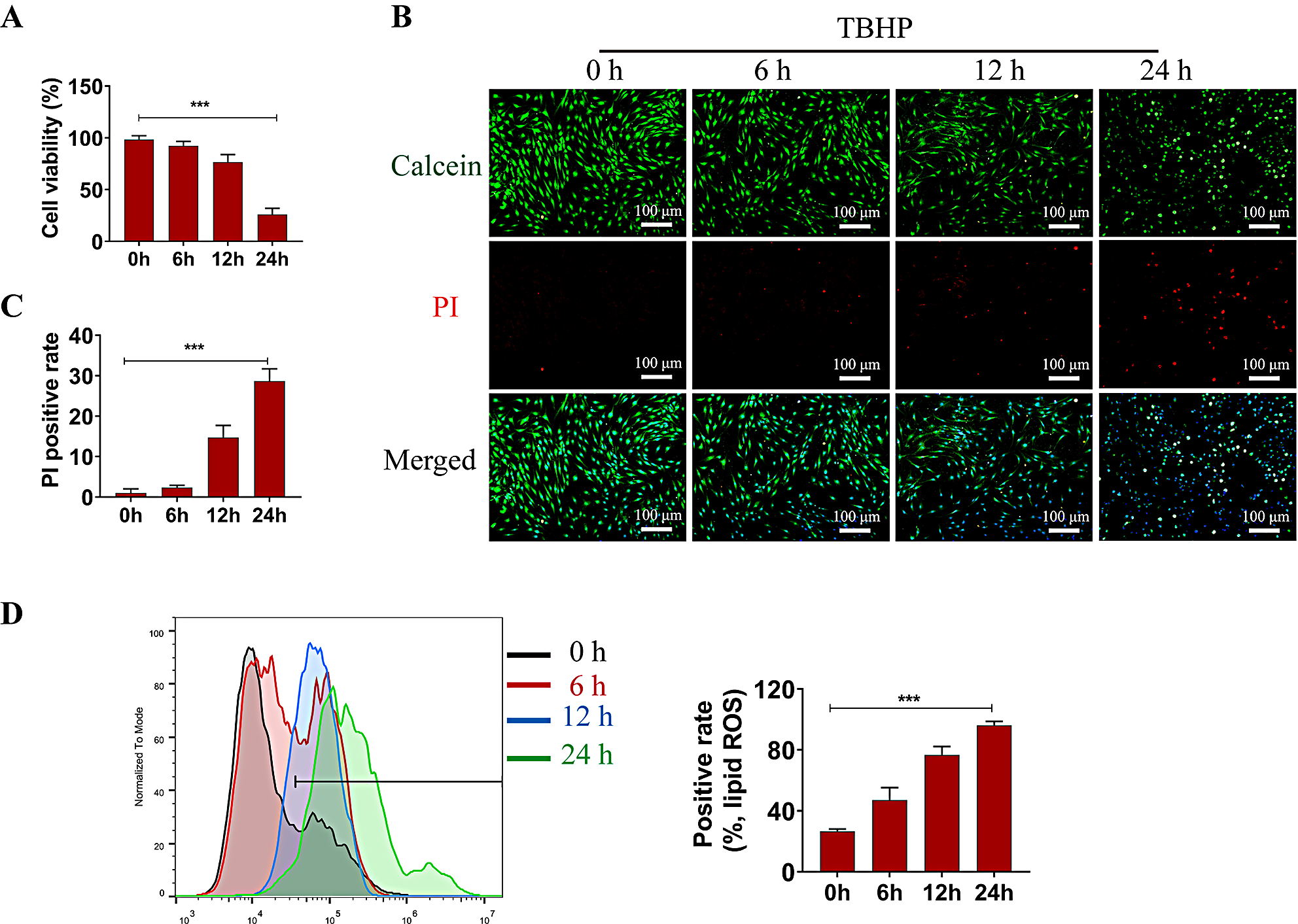


**Figure S2:** TBHP induces the ferroptosis of MSCs in a time-dependent manner. MSCs were treated with TBHP (50 μm) at different time periods (0 h, 6 h, 12 h, 24 h). (A) CCK-8 results of MSCs. (B-C) Live/dead staining of MSCs (B), and the quantitative PI positive rate of total MSCs (C). (D) Lipid ROS level of MSCs (left) and the corresponding quantitative results of lipid ROS level (right). Data were presented as mean ± SD of at least three independent replicates. ****P* < 0.001.


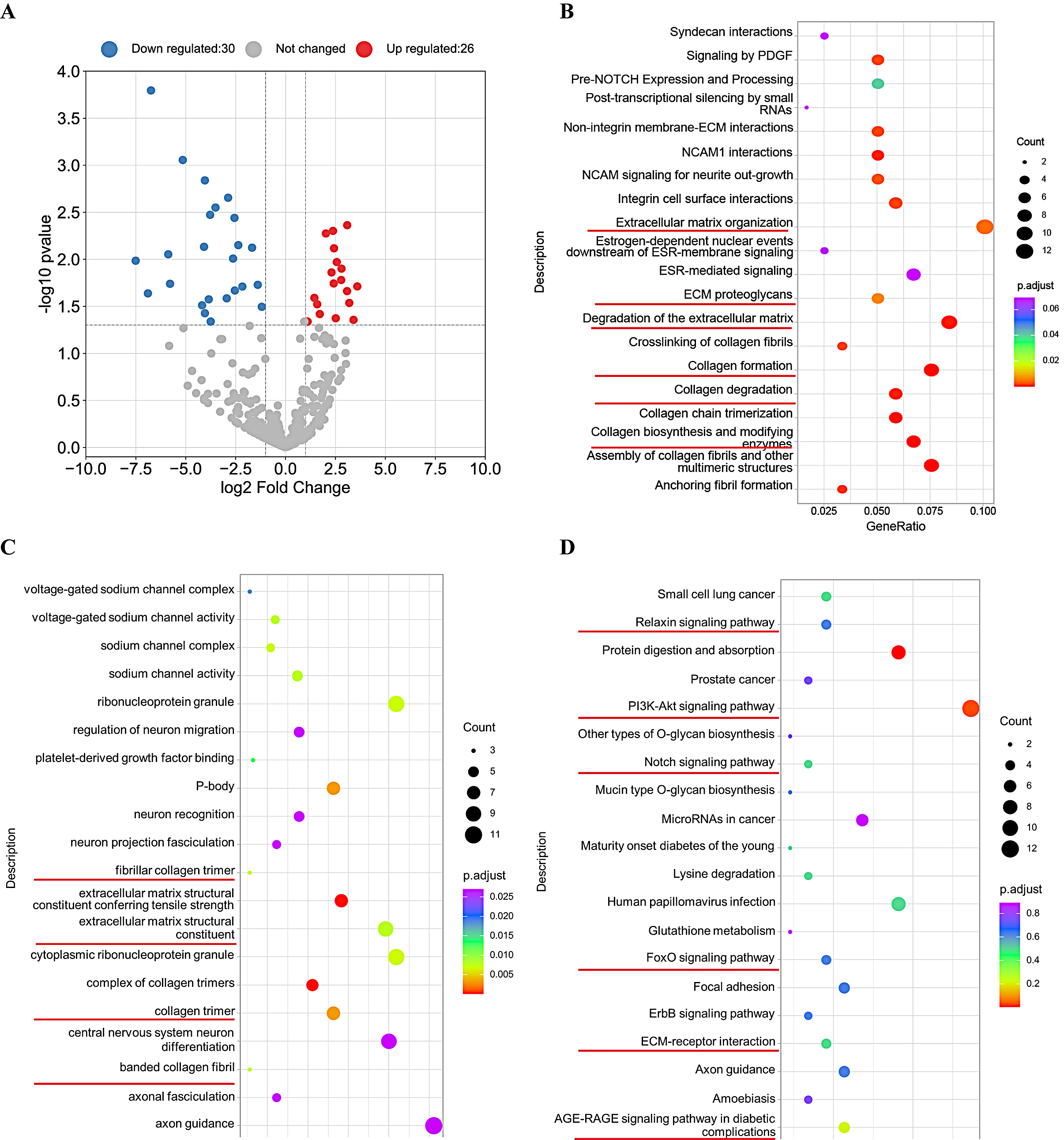


**Figure S3:** Bioinformatic analysis of differential expressed miRNAs in T-sEVs (sEVs derived from TBHP-treated MSCs) vs. C-sEVs (sEVs derived from normal MSCs). (A) Scatter diagram of differential expressed miRNAs. (B) Reactome pathway analysis of differential expressed miRNAs. (C) GO enrichment analysis of differential expressed miRNAs. (D) KEGG pathway analysis of differential expressed miRNAs.


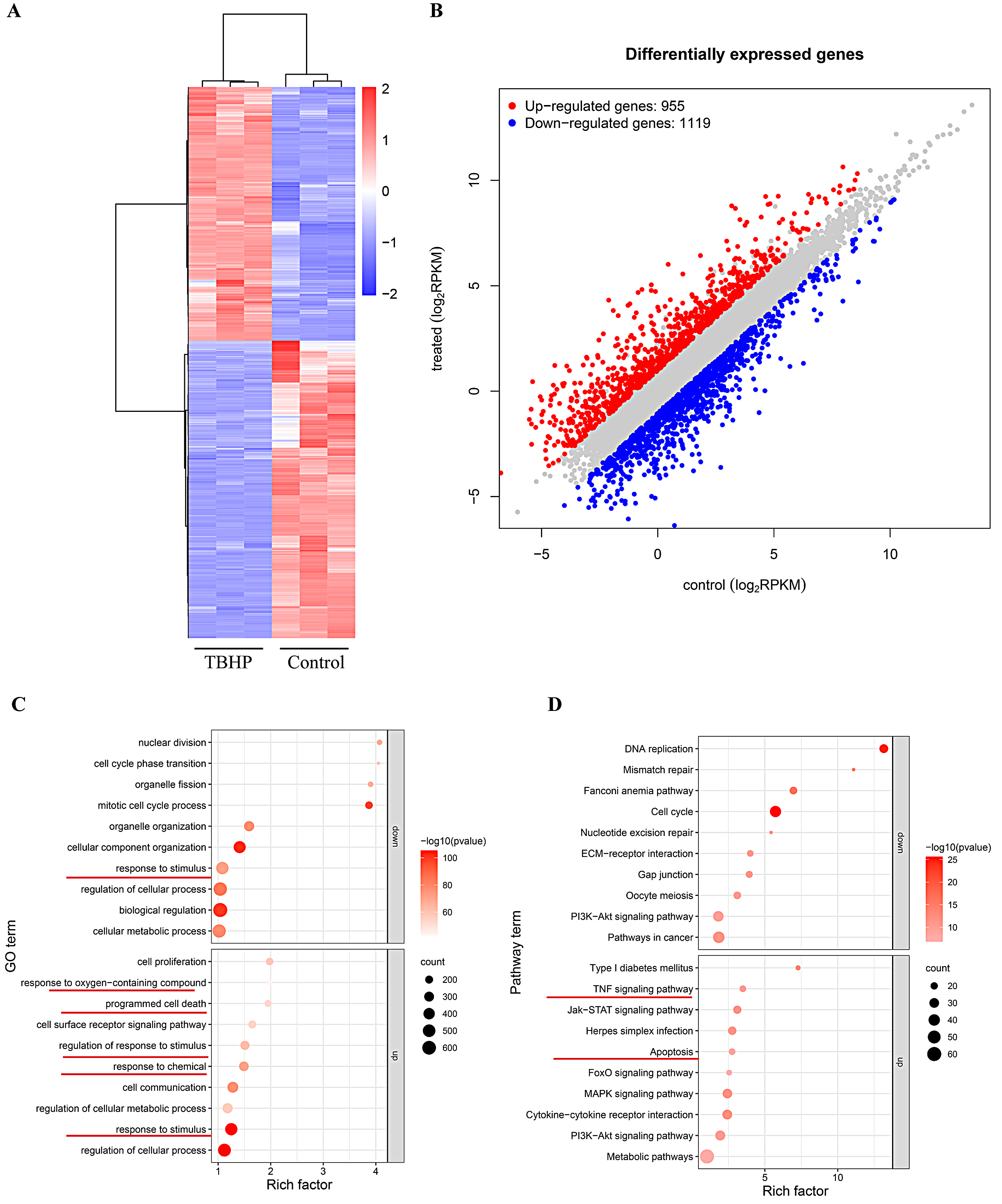


**Figure S4:** Bioinformatic analysis of differential expressed genes in TBHP-treated MSCs vs. normal MSCs. (A) Heatmap of differential expressed genes. (B) Scatter diagram of differential expressed genes. (C) GO enrichment analysis of differential expressed genes. (D) KEGG pathway analysis of differential expressed genes.


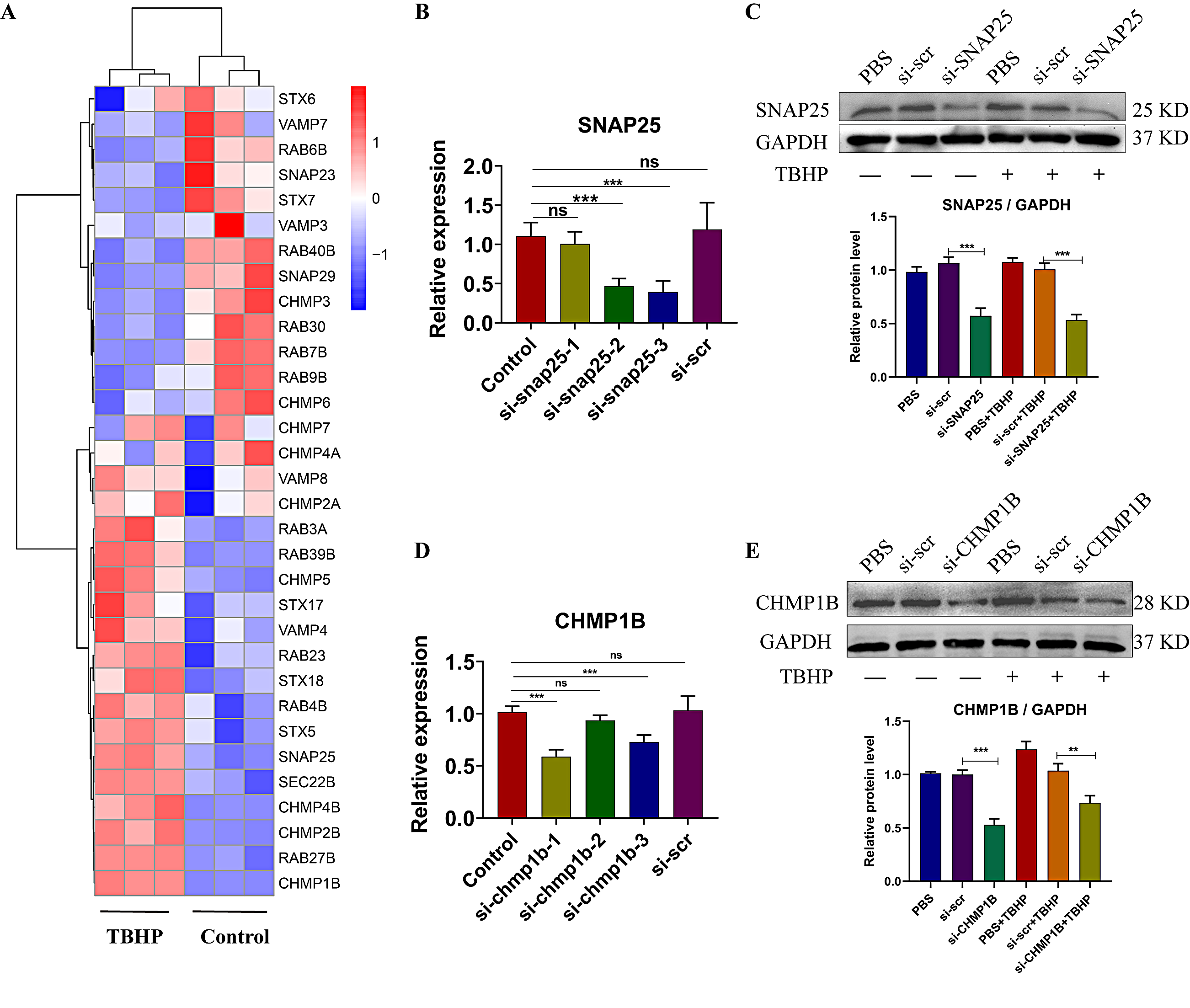


**Figure S5:** Knockdown efficiencies and the colocalization analysis of SNAP25 and CHMP1B. (A) Heatmap of interested differential expressed genes in TBHP-treated MSCs vs. normal MSCs. (B) The mRNA level of SNAP25 in MSCs treated with three siRNAs or a scrambled siRNA (si-scr). (C) The western blot and quantitative protein level of SNAP25 in MSCs treated with selected siRNAs or si-scr. (D) The mRNA level of CHMP1B in MSCs treated with three siRNAs or a scrambled siRNA. (E) The western blot and quantitative protein level of CHMP1B in MSCs treated with selected siRNAs or si-scr. Data were presented as mean ± SD of at least three independent replicates. ***P* < 0.01, ****P* < 0.001, and *ns* for no significant difference.


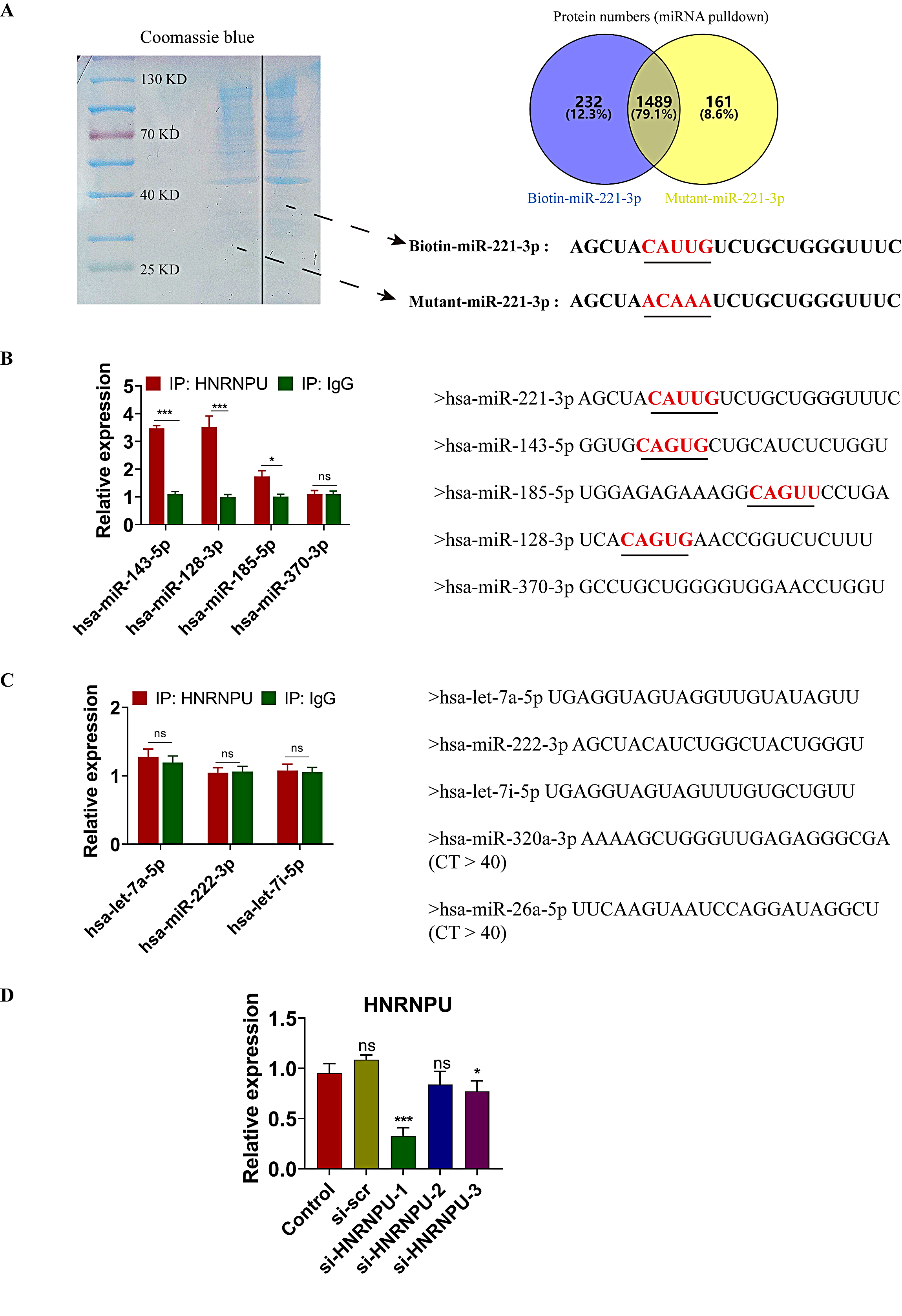


**Figure S6:** HnRNPU participates in the biogenesis of sEVs. (A) Coomassie blue staining of miRNA-precipitated proteins (left). The Venn diagram of precipitated proteins by miR-221-3p and mutant-miR-221-3p (right). (B) RIP analysis of the RNA concentration bound by HNRNPU. The level of 128/143/185/370 precipitated by HNRNPU or IgG was measured. (C) The level of 222/320a or let-7a/7i precipitated by HNRNPU or IgG was measured. (D) The mRNA level of HNRNPU in MSCs treated with three siRNAs or a scrambled siRNA. Data were presented as mean ± SD of at least three independent replicates. **P* < 0.05, ****P* < 0.001, and *ns* for no significant difference.


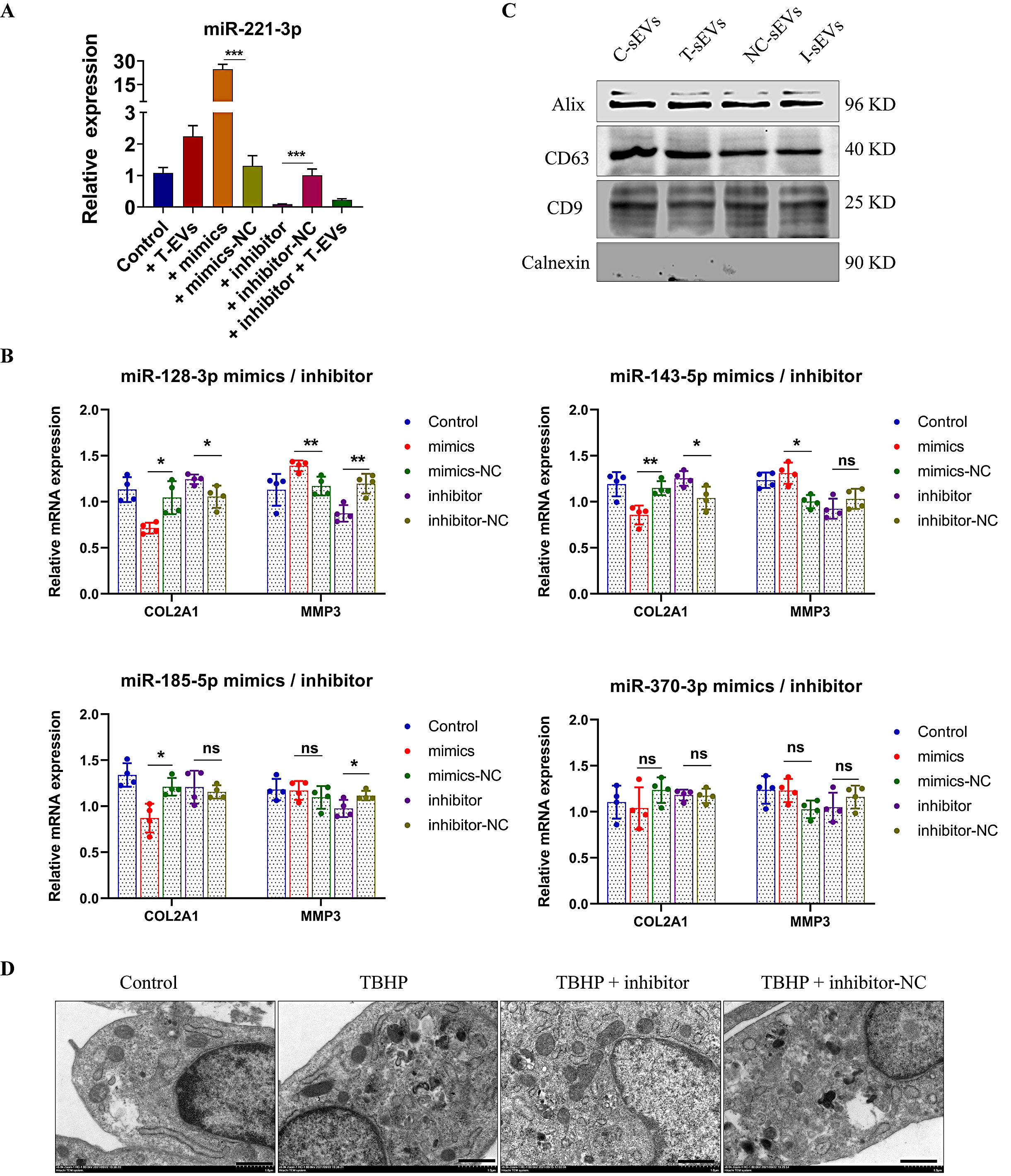


**Figure S7:** Effects of miR-221 inhibition on MSCs. (A) The levels of miR-221-3p in NP cells were measured by RT-qPCR. (B) NP cells were transfected with miR-128/143/185/370 mimics or inhibitors. The mRNA levels of COL2A1 and MMP3 were measured by RT-qPCR. (C) Western blot analysis of Alix, CD63 and CD9 in equivalent C-sEVs (sEVs derived from normal MSCs), T-sEVs (sEVs derived from TBHP-treated MSCs), NC-sEVs (sEVs derived from MSCs transfected with inhibitor-NC) and I-sEVs (sEVs derived from MSCs transfected with miR-221 inhibitor). (D) Representative transmission electron microscopy images of MSCs transfected with miR-221 inhibitor or inhibitor-NC. **P* < 0.05, ***P* < 0.01, ****P* < 0.001, and *ns* for no significant difference.

**
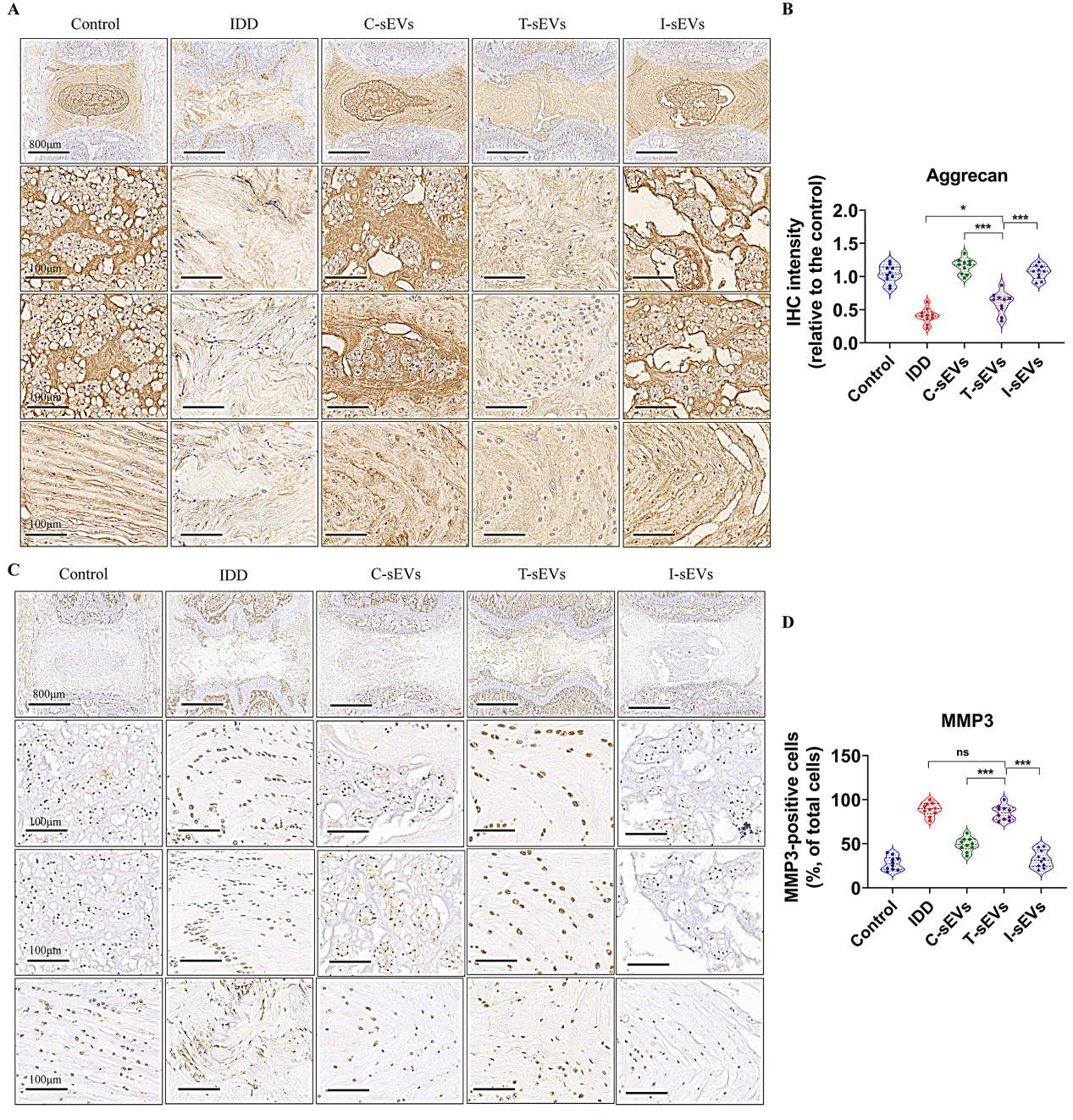
**

**Figure S8:** MiR-221 inhibition alters the effects of sEVs on IDD *in vivo*. (A-B) Immunohistochemistry staining of aggrecan in discs treated with different sEVs (A), and the quantitative results based on the mean IHC intensity (B). (C-D) Immunohistochemistry staining of MMP3 in discs treated with different sEVs (C), and the quantitative results of MMP3-positive cell rate (D). Data were presented as mean ± SD of at least three independent replicates. **P* < 0.05, ****P* < 0.001, and *ns* for no significant difference.

**Supplemental Tables (S1-S3)**

| **Table S1：Primary antibodies** | | | | | |
| --- | --- | --- | --- | --- | --- |
| Name | Type | Source | Address | Catalog number | Application |
| Anti-GPX4 | Polyclonal | Affinity | OH, USA | DF6701 | WB |
| Anti-FPN | Polyclonal | Affinity | OH, USA | DF13561 | WB |
| Anti-FTL | Polyclonal | Proteintech | Wuhan, China | 10727-1-AP | WB |
| Anti-DMT1 | Polyclonal | Proteintech | Wuhan, China | 20507-1-AP | WB |
| Anti-CHMP1B | Polyclonal | Proteintech | Wuhan, China | 14639-1-AP | WB, IF, IP |
| Anti-CD63 | Monoclonal | Proteintech | Wuhan, China | 67605-1-Ig | WB, IF |
| Anti-CD9 | Monoclonal | Proteintech | Wuhan, China | 60232-1-Ig | WB, IF |
| Anti-HNRNPU | Polyclonal | Proteintech | Wuhan, China | 14599-1-AP | WB, IF, IP, RIP |
| Anti-Alix | Polyclonal | Proteintech | Wuhan, China | 12422-1-AP | WB |
| Anti-Calnexin | Polyclonal | Proteintech | Wuhan, China | 10427-2-AP | WB |
| Anti-GAPDH | Monoclonal | Proteintech | Wuhan, China | 60004-1-Ig | WB |
| Anti-Lamin B1 | Monoclonal | Proteintech | Wuhan, China | 66095-1-Ig | WB |
| Mouse IgG | — | Proteintech | Wuhan, China | B900620 | WB, IP |
| Rabbit IgG | — | Proteintech | Wuhan, China | B900610 | WB, IP |
| Anti-SNAP25 | Monoclonal | Abcam | Cambridge, UK | ab66066 | WB, IF, IP |
| Anti-p-SNAP25 | Polyclonal | Abcam | Cambridge, UK | ab169871 | WB |
| Anti-Collagen II | Polyclonal | Servicebio | Wuhan, China | GB111629 | IHC |
| Anti-Aggrecan | Polyclonal | Servicebio | Wuhan, China | GB11373 | IHC |
| Anti-MMP3 | Polyclonal | Servicebio | Wuhan, China | GB11131 | IHC |
| Anti-MMP13 | Polyclonal | Servicebio | Wuhan, China | GB11247 | IHC |
| Ab. WB, western blot; IF, immunofluorescence; IP, immunoprecipitation; RIP, RNA immunoprecipitation; IHC, immunohistochemistry. | | | | | |

| **Table S2：Primers of targeted genes** | | |
| --- | --- | --- |
| Name | Forward | Reverse |
| SNAP25 | TGGCCTTTTCATATGTCCTT | CATCCATTTCATTTTCTCGG |
| CHMP1B | TCGATGGCTGGTGTGGTTA | TGGTTCTGGGGAGTGGTGA |
| RAB27B | TAGACTTTCGGGAAAAACGTGTG | AGAAGCTCTGTTGACTGGTGA |
| CHMP5 | AGATTTCTCGATTGGATGCTGAG | TGTTGGGCAAGATTGTCCCG |
| CHMP4B | TGCAGAGGAGATTTCAACAGC | TGTTTCGGGTCCACTGATTTC |
| SEC22B | CCGGGACCTTCAACAATATCAG | GCAAAAGCCAACTTCTTAGGGA |
| COL2A1 | AGAACTGGTGGAGCAGCAAGA | AGCAGGCGTAGGAAGGTCAT |
| ACAN | TGAGCGGCAGCACTTTGAC | TGAGTACAGGAGGCTTGAGG |
| MMP3 | TTCCTTGGATTGGAGGTGAC | AGCCTGGAGAATGTGAGTGG |
| MMP13 | CCCAACCCTAAACATCCAA | AAACAGCTCCGCATCAACC |
| HNRNPU | GAGCATCCTATGGTGTGTCAAA | TGACCAGCCAATACGAACTTC |
| β-ACTIN | CCTTCCTGGGCATGGAGTC | TGATCTTCATTGTGCTGGGTG |
| hsa-miR-221-3p | CCGAGAGCTACATTGTCTGCTG | AGTGCAGGGTCCGAGGTATT |
| hsa-miR-370-3p | AGCCTGCTGGGGTGGAA | AGTGCAGGGTCCGAGGTATT |
| hsa-miR-143-5p | GGGGTGCAGTGCTGCATC | AGTGCAGGGTCCGAGGTATT |
| hsa-miR-185-5p | CCGAGTGGAGAGAAAGGCAGT | AGTGCAGGGTCCGAGGTATT |
| hsa-miR-128-3p | CCGAGTCACAGTGAACCGGT | AGTGCAGGGTCCGAGGTATT |
| hsa-let-7a-5p | CCGAGTGAGGTAGTAGGTTG | AGTGCAGGGTCCGAGGTATT |
| hsa-miR-222-3p | GGGAGCTACATCTGGCTAC | AGTGCAGGGTCCGAGGTATT |
| hsa-let-7i-5p | CCGAGTGAGGTAGTAGTTTGT | AGTGCAGGGTCCGAGGTATT |
| hsa-miR-320a-3p | GGGAAAAGCTGGGTTGAGA | AGTGCAGGGTCCGAGGTATT |
| hsa-miR-26a-5p | CCGAGTTCAAGTAATCCAGGA | AGTGCAGGGTCCGAGGTATT |
| U6 | AGCACATATACTAAAATGGAACGAT | AGTGCAGGGTCCGAGGTATT |
| Name | RT primer | |
| hsa-miR-221-3p | GTCGTATCCAGTGCAGGGTCCGAGGTATTCGCACTGGATACGACGAAACC | |
| hsa-miR-370-3p | GTCGTATCCAGTGCAGGGTCCGAGGTATTCGCACTGGATACGACACCAGG | |
| hsa-miR-145-5p | GTCGTATCCAGTGCAGGGTCCGAGGTATTCGCACTGGATACGACACCAGA | |
| hsa-miR-185-5p | GTCGTATCCAGTGCAGGGTCCGAGGTATTCGCACTGGATACGACTCAGGA | |
| hsa-miR-128-3p | GTCGTATCCAGTGCAGGGTCCGAGGTATTCGCACTGGATACGACAAAGAG | |
| hsa-let-7a-5p | GTCGTATCCAGTGCAGGGTCCGAGGTATTCGCACTGGATACGACAACTAT | |
| hsa-miR-222-3p | GTCGTATCCAGTGCAGGGTCCGAGGTATTCGCACTGGATACGACACCCAG | |
| hsa-let-7i-5p | GTCGTATCCAGTGCAGGGTCCGAGGTATTCGCACTGGATACGACAACAGC | |
| hsa-miR-320a-3p | GTCGTATCCAGTGCAGGGTCCGAGGTATTCGCACTGGATACGACTCGCCC | |
| hsa-miR-26a-5p | GTCGTATCCAGTGCAGGGTCCGAGGTATTCGCACTGGATACGACAGCCTA | |
| U6 | GTCGTATCGACTGCAGGGTCCGAGGTATTCGCAGTCGATACGACAAAATATG | |

| **Table S3: List of siRNA or miRNA sequences** | |
| --- | --- |
| Name | Sequence (5’→3’) |
| si-SNAP25: #1, -234 | GCAAUGAGCUGGAGGAGAUTT |
| si-SNAP25: #2, -361 | GGAUGAACAAGGAGAACAATT |
| si-SNAP25: #3, -714 | UGUGUAUCGAUCUCAUUGCTT |
| si-CHMP1B: #1, -230 | ACAUGGAAGUUGCGAGGAUTT |
| si-CHMP1B: #2, -443 | UGGACAAAUUCGAGCACCATT |
| si-CHMP1B: #3, -533 | CCCAGAACCAAGUGGAUAUTT |
| si-HNRNPU: #1, -230 | CGGACAAUUACAUUUUUUCTT |
| si-HNRNPU: #2, -926 | ACCUAUAAAACUCAUGUAATT |
| si-HNRNPU: #3, -954 | UUCAUAUCGUCUCGGUUUATT |
| si-scr | UUCUCCGAACGUGUCACGUTT |
